# Study Protocol of “Sustainably Healthy—From Science 2 Highschool & University”—Prevalence of Mixed, Vegetarian, and Vegan Diets Linked to Sports & Exercise among Austrian Tertiary Students and Lecturers/Academic Staff

**DOI:** 10.3390/ijerph192215313

**Published:** 2022-11-19

**Authors:** Katharina C. Wirnitzer, Mohamad Motevalli, Derrick R. Tanous, Gerold Wirnitzer, Karl-Heinz Wagner, Armando Cocca, Manuel Schätzer, Werner Kirschner, Clemens Drenowatz, Gerhard Ruedl

**Affiliations:** 1 Department of Research and Development in Teacher Education, University College of Teacher Education Tyrol, 6010 Innsbruck, Austria; 2 Department of Sport Science, University of Innsbruck, 6020 Innsbruck, Austria; 3 Research Center Medical Humanities, Leopold-Franzens University of Innsbruck, 6020 Innsbruck, Austria; 4 AdventureV & Change2V, 6135 Stans, Austria; 5 Department of Nutritional Sciences and Research Platform Active Ageing, University of Vienna, 1090 Vienna, Austria; 6 Special Institute for Preventive Cardiology and Nutrition—SIPCAN, 5061 Elsbethen, Austria; 7 Division of Sport, Physical Activity and Health, University of Teacher Education Upper Austria, 4020 Linz, Austria

**Keywords:** emerging adulthood, lifestyle, physical activity, dietary behavior, diet type, plant-based, sustainable diet, conventional diet, public health

## Abstract

Healthy lifestyle is the major indicator of individual and public health especially in target populations (e.g., during emerging adulthood). Evidence indicates that unhealthy lifestyle behaviors are strongly associated with the increasing prevalence of chronic diseases. The dual approach of sustainable health (i.e., physical exercise tied to a healthy diet) is an effective lifestyle strategy to control and manage health-related disorders, including overweight/obesity. Based on the evidence on plant-based diets regarding health and considering the growing prevalence of vegans and vegetarians worldwide, limited data exist on the health-related behaviors of those who follow plant-based vs. mixed diets in young adulthood. This multidisciplinary study is the first to examine the association between diet type (mixed, vegetarian, and vegan diets) and various health-related behaviors (primarily physical activity, sport, & exercise) among college/university students and lecturers/academic staff in Austria nationwide. Following a cross-sectional study design and using online questionnaires, 4510 tertiary students and 1043 lecturers/academic staff provided data on sociodemographic characteristics, dietary patterns, physical exercise habits, and other lifestyle behaviors (sleep, alcohol intake, smoking, etc.) along with information on health status and quality of life. While the data will be analyzed based on differentiated sociodemographic and health-related categories, the influence of the COVID-19 pandemic on lifestyle behaviors will also be evaluated. As a study protocol, this article represents comprehensive details of the design, purposes, and associated analytical measures of the present study within the tertiary educational context.

## 1. Introduction

A healthy lifestyle is one major contributor to individual and public health, while unhealthy lifestyle behaviors are associated with various health problems, including non-communicable diseases (NCDs) [1,2,3,4]. Recent trends worldwide show a high prevalence of unhealthy lifestyle choices and excess body weight worldwide [5,6] that contribute remarkably to massive prevalence of chronic cardio-metabolic disorders (e.g., hypertension, hyperglycemia, dyslipidemia), different types of cancers, and several psychosocial problems (e.g., discrimination, social isolation, low self-esteem) [5,7,8]. While NCDs are accountable for 74% of deaths worldwide [3], research indicates that most cases are preventable and even reversible [9,10].

It has been well-documented that the prevention and management of most health conditions are greatly influenced by different lifestyle factors [1,11,12,13]. As a substantial part of a healthy lifestyle, physical activity (PA), including regular engagement in sports & exercise, is considered an independent (or even a supplementary) “medicine” for several health problems [9,10,14,15], including overweight and obesity [16,17]. However, PA is not enough to promote sustainable health without the support of other healthy behaviors; therefore, integrating lifestyle factors, particularly the combination of PA with a sustainably healthy diet, leads to greater health benefits due to the cumulative effects [1,18,19,20,21]. The effectiveness of the well-accepted dual approach of “Healthy Eating, Active Living” [22,23] has been concluded by various systematic reviews investigating health and weight management approaches, which suggests that the combination of PA and nutritional strategy is likely to yield better outcomes than single-component strategies [24,25,26,27]. Additionally, the interaction of diet and PA in the regulation of energy balance highlights their reciprocal role in weight management and health [28,29]. However, nutritional patterns are not limited to the concept of energy balance. It is well-established that the most appropriate nutritional strategy to achieve long-term metabolic and mental health is shifting to healthy food choices [30], particularly relying on more whole-food plant-based diets [31,32,33].

A vegetarian/vegan diet, containing whole plant foods specifically, is considered a sound recommendation for long-term health and recognized as “medicine” when applied to health conditions [34,35,36,37,38] and may protect against moderate-to-severe COVID-19, as well [39,40,41,42,43]. According to the epidemiological data, the prevalence of plant-based diets is growing worldwide at a faster rate than the expectations [44,45]. Reports show that 10% of Europeans (~75 million) adhere to a vegan or vegetarian diet [46], with ~2% greater rate in German-speaking countries (i.e., Germany, Austria, Switzerland) than the European average [47,48]. A more noticeable increasing trend toward plant-based diets [49,50] and a higher prevalence of vegan and vegetarian diets [51,52,53] have been reported in younger adults. Interestingly, it has been reported that the number of vegans and vegetarians has doubled in Germany since the beginning of the COVID-19 pandemic [54]. Based on the recent trends toward plant-based diets, consumers are increasingly demanding meat-free meals and dishes. This major dietary shift has resulted in considerable changes in the food offerings of supermarkets, restaurants, and hotels (snack bars, fast food chains, dormitories, etc.), private and public catering, and buffets and canteens (kindergartens, schools, colleges/universities, hospitals, senior residences, jails, etc.) during the past years offering new varieties of vegan and vegetarian options [45,55].

Although developing NCDs and their associated risk factors (including overweight/obesity) can potentially begin as early as in utero [56], research shows that they mainly develop over the lifespan, posing the greatest threat to health and wellbeing [4,57]. According to the literature available, emerging adulthood, defined as the period between the late teens and early twenties [58], is a critical period to shape lifestyle patterns and consequential health-related (including body weight) outcomes [59,60,61]. Despite there being some evidence indicating a high prevalence of unhealthy lifestyle behaviors (insufficient PA and poor dietary habits) [62,63,64], the social environment, especially during the pandemic of COVID-19, is considered a critical contributor to the establishment of health behaviors in university students [65]. Therefore, since both university life and emerging adulthood are linked to psychological stressors that undesirably affect health and future life [66], serious consideration in the dual approach must always be given in health-related educational policy in order to enhance health literacy and, consequently, strengthen the opportunities for sustainable health behaviors across emerging adulthood. Hence, this period in which individuals have left behind the relative dependency of childhood/adolescence but have not yet taken on the responsibilities of adulthood [60,67], is crucial to cultivate and/or develop health behaviors and habits that potentially lead to a sustainable and lifelong adherence to a healthy lifestyle.

Considering the involvement of various populations (families, children, health and education experts, scientists, decision makers, political authorities, etc.), there is an overarching responsibility for health with the recommendation to include major participatory input at all levels of society [68]. According to the well-established fact declared by the United Nations Sustainable Development Goals (UN SDGs; especially No. 3 and 4), the WHO Voluntary Global Targets on NCDs (No. 3 in particular), and the UNESCO Learning Objective “Cross-cutting Key Competencies”, health is one of the major topics for human development and satisfaction; thus, it is an overarching objective for educational entities and policymakers to consolidate health into education and curricula [69,70,71]. The concept of health education raises the claim of holistic personality development with the background of health-oriented action competence and sustainable willingness to act [72]. Health-threatening behaviors, seen especially in socially disadvantaged populations with limited access to educational programs, are less common in populations with higher educational backgrounds, especially in people with knowledge, skills, and competencies considering healthy lifestyle [73,74,75,76]. Therefore, educational settings such as schools and universities can provide a suitable environment for health behavior interventions and for developing health behaviors [77,78,79,80], which can influence the lifestyle choices of pupils and students independent of their socioeconomic backgrounds [78,79].

Scientific data on the health behaviors of Austrian populations are limited, particularly on college/university students [81,82]. While the advantages of disease prevention by adhering to a healthy lifestyle are well documented, current policies targeting overweight and obesity are mainly based on therapeutic approaches rather than preventative strategies [83]. In 2017, Austria spent EUR 45.4 billion on healthcare, and those costs are expected to increase by 4.9% annually [84]. These preventable costs are in line with the increasing prevalence of overweight/obesity in Austrian adults, which is currently more than 50% and reported to be higher in women than men and in Eastern Austria compared with Western Austria [5,81,85,86]. Evidence reports that more than half of Austrian adults (54%) exceed the reference values for energy intake [87], and a comparable percentage (53%) do not meet the amount of recommended PA [88,89]. Data from another study show that 34% of adults in Austria suffer from lifestyle-related chronic health conditions [90]. There remains, however, limited information on the current PA and nutrition trends of Austrian college and university students [91]. Results from a study showed that 60%, 47%, and 30% of university students had insufficient PA, unbalanced diet, and low mental well-being, respectively [63], and the majority (9 out of 10) of predictors of unhealthy lifestyle behaviors in university students were environmental or institutional barriers [63]. Data show that the COVID-19 pandemic and social lockdowns have further restricted health behaviors, with PA opportunities particularly affected [92,93,94]. A 22% increase in the number of adults having gained weight was reported as a result of unhealthy lifestyle behaviors during the COVID-19 pandemic, mainly resulting from decreased PA levels and overeating [92]. Regardless of the direct effect of the recent pandemic on lifestyle behaviors (particularly PA and diet), it has been shown that there is an association between obesity and many viral pandemics, including the 2009 swine flu [95] and COVID-19 [96], which has uncovered new insights regarding mortal characteristics of obesity and unhealthy lifestyle behaviors.

The importance of monitoring and prioritizing effective strategies to improve university student health has been highly emphasized in the literature [62,63,64]. Research-based evidence in public health provides the basis for governments and health organizations to establish their policies and guidelines. Particularly, efforts towards enhancing the understanding of the interaction between PA and diet type during the transition from childhood to adulthood is crucial for health-oriented purposes, especially in the wake of the COVID-19 situation. Hence, investigating lifestyle behaviors by dietary subgroups and analyzing various interactions with sociodemographic aspects (e.g., sex, age, Body Mass Index (BMI), and living area) seems crucial to expand the current understanding of the unenthusiastic health status among young adult populations.

To date, there is no data about the association between diet type and lifestyle behaviors (particularly PA, sports & exercise) in Austrian college/university students and lecturers/academic staff. There is only one Austrian study that has been conducted in the secondary educational setting focusing on dietary trends (vegetarian and vegan vs. mixed diets) linked to PA patterns, entitled “From Science 2 School: Sustainably healthy—active & veggie” (S2S) [97,98,99]. To seamlessly extend the previous research on secondary levels I and II to the advanced education level, the current and crucial follow-up study was designed in the Austrian tertiary educational setting.

## 2. Objectives and Hypotheses

The aim of the present multidisciplinary college and university study, *Sustainably healthy—From Science 2 Highschool & University* (S2HU; www.uni.science2.school/en/, accessed on 16 November 2022), is to survey and examine, for the first time, the prevalence of mixed (traditional) and plant-based (sustainable) diets in connection with several lifestyle behaviors, including sports & exercise, alcohol intake, smoking, sleep patterns, etc., among Austrian college and university students and lecturers/academic staff with the following goals:

1. The first and major goal of this college/university study is to survey the prevalence of omnivorous, vegetarian, and vegan diets linked to levels of sports & exercise;
2. The second goal is to investigate the health-related behaviors of adults but particularly those of emerging adulthood (students) at tertiary educational entities;
3. The third goal is to validate the self-reported data regarding lifestyle habits with a special focus on diet type and engagement in sports & exercise.

Taking a large sample into account, S2HU is the first study to examine the relationship between sports & exercise and diet type in the context of tertiary education. This study aims to add current information to the dietary tend data available on adults. This work is necessary to expand the epidemiological knowledge in adult populations—with a special focus on emerging adulthood—particularly on students and academic staff within the tertiary educational setting. Based on sound theory, population and cohort studies have shown increased health consciousness and healthier lifestyles among people/adults following some kind of plant-based diet (but especially the vegan diet) rather than those following an omnivorous diet [100,101]. This theory is based on the avoidance of unhealthy practices (as well as appropriate levels of rest and relaxation) with the vegan sub-population being significantly more physically active and less likely to smoke or drink alcohol [102].

Therefore, it was hypothesized that vegetarian (but especially vegan) students and lecturers/academic staff show a healthier lifestyle (higher engagement in sports & exercise, for example, among other lifestyle parameters).

## 3. Materials and Methods

### 3.1. Study Design

*Sustainably healthy—From Science 2 Highschool & University* was designed as a seamless follow-up to the original S2S study. In order to address the gap in the scientific literature for S2HU, the S2S objectives and questions were transferred to the tertiary education level and expanded regarding the corresponding settings (college, university) to generate consistent results into (emerging) adulthood. The present interdisciplinary project (Sport Science, Nutrition Science, Health Science) follows a cross-sectional design based on a multi-level cluster sampling strategy, similar to the previous school study [97]. An official website (https://uni.science2.school/en, accessed on 16 November 2022) was specifically set up with the purpose of providing full information on the present study.

*Epidemiology*. The survey was conducted in Austria, nationwide, to collect data on demography (nationality, age, sex, federal state, and place of residence: urban vs. rural region); biometric data (height, body weight, and calculated BMI); marital status; highest academic qualification; current health status; professional role (students vs. lecturer/academic staff): students (study: bachelor, master, or Ph.D., respective semester, field of study, teacher/pedagogy–didactics training program/specialized sciences) and lecturer/academic staff (employment and extent, type of college/university, subject area/field of activity); current physical exercise behaviors (motives, type of summer/winter sports; duration/day, frequency/week, competition participation, member of sports club); current dietary adherence (kind of diet; motivation and duration of current diet; buffet/canteen offer; daily fluid intake; consumption of fruit and vegetables considering frequency and number of portions; which meals; self-assessment of one’s own macronutrient distribution); individual assessment of lifestyle factors that promote health and wellbeing (sports & exercise: frequency; duration, intensity; food: animal, plant, supplements; sleep: duration and quality on work days/weekends; other factors: individually or in combination) [1,13]; food/ingredient allergies/intolerances; health status, including chronic/serious illness; quality of life WHO-QOL-BREF-26 (permission by 6. 8. 2020) [103]; and independent data on lifestyle and behavioral changes during the COVID-19 pandemic.

### 3.2. Sample

In Austria, there are 102 colleges/universities with 376,050 students and 69,310 lecturers, which results in a total sample size of approximately 445,360 potential participants. It was planned to include as many study participants as possible in order to obtain the most meaningful results possible with the support of a large and representative data set. The target group was all students and lecturers/academic staff established in their respective professional roles at an Austrian tertiary educational entity and/or university.

The study was initiated (on 5 April 2021) by: (1) contacting the college/university management and the board of deans by e-mail or directly by personal communication (e.g., telephone call) to give detailed information about the aim and study procedure and to share the link for the online survey; and (2) instructions were provided with relevant information for student and lecturer/academic staff survey participation. The survey was available to complete individually (also at home) or as a didactic element in courses during regular class sessions (students together with the course directors), whereby the college/university management and board of deans were also invited to participate in the survey. At the closure of data collection (31 July 2021), 6148 students and 1455 lecturers/academic staff filled in the online survey. From those respondents, 4510 students (1.2% of the eligible 376,050) and 1043 lecturers/academic staff (1.5% of 69,310 eligible sample) provided complete data sets. Therefore, the previously anticipated response rate of 1.0% of the total potential Austrian sample was successfully achieved.

### 3.3. Inclusion Criteria

Any participant who attended a tertiary educational entity or was employed in an official capacity of a college or university could participate, including students and lecturers/academic staff at Austrian colleges/universities. For successful participation and to include participant data in the study analyses, a complete data set consisting of a comprehensively filled-out questionnaire (1) and the written declaration of consent and publication (2) was required.

### 3.4. Questionnaire

The standardized online questionnaire was provided in German (LimeSurvey, version 3.25.15) in six parts with questions about the person (Part A), sports & exercise (Part B), nutrition (Part C), health & well-being (Part D), COVID-19 (Part E), and miscellaneous (Part F) [1,44,97,102,103,104,105,106,107,108,109,110,111,112,113,114,115,116,117,118,119,120,121,122,123,124,125].

Different types of control questions were included in the online questionnaire to identify contradictory information to obtain the most reliable data possible. Study participation was voluntary and completing the online survey took about 20 min.

The survey for this college/university study was based on self-report or self-assessment. All study participants accessed the questionnaire via an encrypted interface by a web link and filled it out conveniently and easily with a smartphone, tablet, or PC/laptop. The online form was designed to distinguish between students and lecturers/academic staff within the survey (see Supplementary Material). In addition, information on the survey in the context of the study was also available on the official website (https://uni.science2.school/en/, accessed on 16 November 2022), which was created specifically for this purpose.

**Figure 1:**
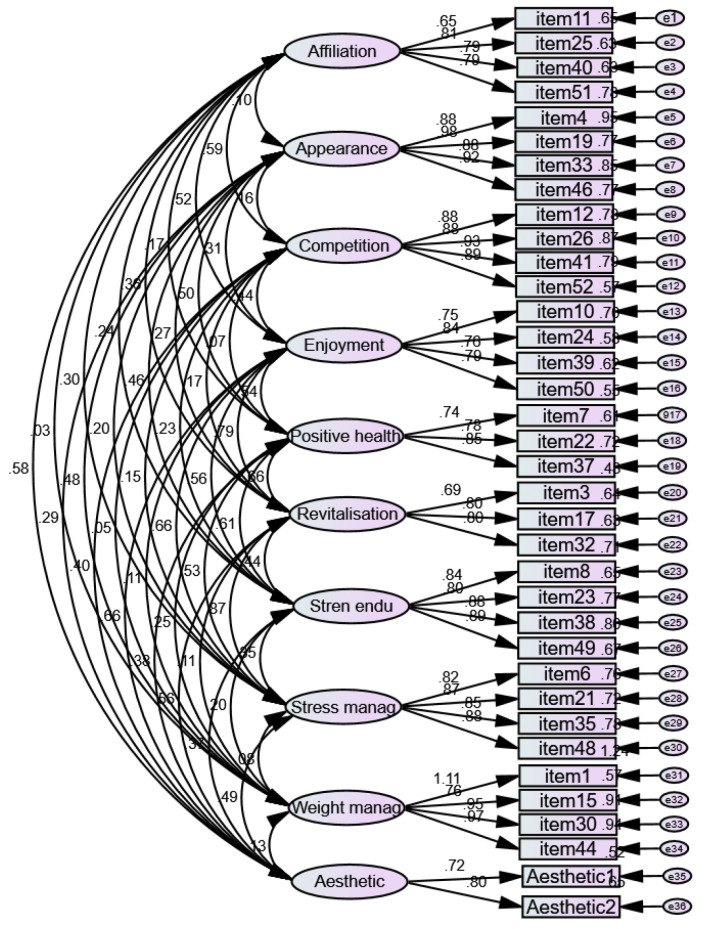
Structural model of the Exercise Motivations Inventory (EMI-2). Note: Stren endu = strength and endurance; Stress manag = stress management; and Weight manag = body weight management.

*Validation of Questionnaire*. The present survey was developed based on the relevant literature [1,44,97,102,103,104,105,106,107,108,109,110,111,112,113,114,115,116,117,118,119,120,121,122,123,124,125], particularly from validated questionnaires [109,110,115,120,122,123], well-known large-size studies/reports [107,118,120,124], and recognized literature of renowned authors [102,125]. Accordingly, participants were asked to respond mainly to single-choice items, multiple-choice items, and their preferences among several options (such items cannot be grouped to describe latent variables; therefore, no content, construct, or criterion-related validity could be assessed for them, and they should be individually considered when analyzing and interpreting the data). Part B Section 2 (Supplementary Material) of the questionnaire was developed using an existing and previously validated questionnaire, the Exercise Motivations Inventory (EMI-2; Figure 1) [109,122,123], and the German version is available from the official instrument website [126]. The version implemented in this work included 36 items grouped into 10 sub-scales: affiliation (4 items), appearance (4 items), competition (4 items), enjoyment (4 items), positive health (3 items), revitalization (3 items), strength and endurance (4 items), stress management (4 items), weight management (4 items), and aesthetics (2 items). Due to the characteristics of the EMI-2, confirmation of its structure was assessed for the data collected in our study. Confirmatory analysis was carried out with AMOS 22 software (IBM, Chicago, IL, USA) and aimed to test the validity of the 10-subscale model (Figure 1). The outcomes of the validation process showed optimal psychometric parameters (χ^2^ = 8710.399; df = 517; CFI = 0.95; SRMS = 0.505) in agreement with the recommendations made by Hu and Bentler, 1999, [127] on model fit assessment. Internal consistency for the ten subscales ranged from α = 0.765 to α = 0.942.

### 3.5. Procedure and Trial Status

**Figure 2:**
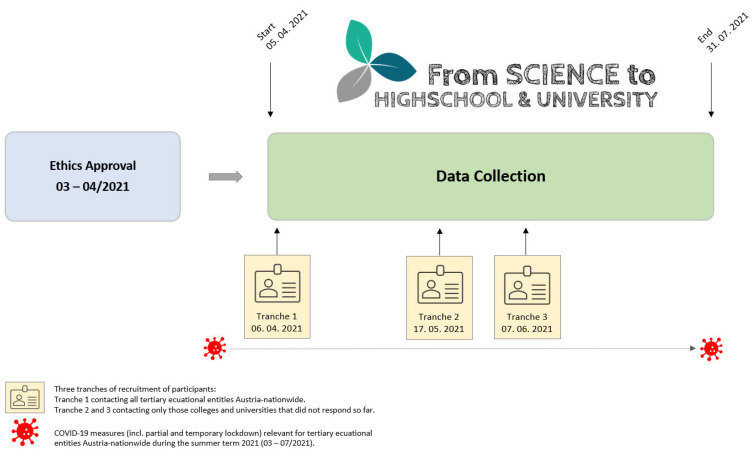
Flow chart of the Austria-nationwide procedure and timescale of the online survey (ethics vote, participant recruitment, and subsequent data collection).

Figure 2 displays the timescale of the preceding study procedure from approval of the lead agencies (PHT—University College of Teacher Education Tyrol and LFUI—Leopold Franzens University of Innsbruck) to the subsequent participant recruitment and the closure of data collection. Regarding the operational implementation, two steps were initially required: (1) support from the Federal Ministry of Education, Science, and Research to facilitate contact/communication with the participating tertiary education entities (which also aided in reaching the highest possible response rate for a representative data set); and (2) ethics approval to conduct the survey directly at all colleges and universities in Austria (nationwide) with the respective college/university management.

Data collection for students and lecturers/academic staff was open during the summer semester of 2021, explicitly from 5 April through 31 July 2021 (Figure 2). Data analyses and the associated interpretation of findings were completed subsequent to the closure of data collection.

### 3.6. Outcome Measures

#### 3.6.1. Primary Outcome Measures

The main outcome is the prevalence of PA, sports & exercise, and the prevalence of kinds of diet (mixed, vegetarian, and vegan), as well as the linkage of activity level and diet type among students and lecturers/academic staff at the Austrian tertiary educational setting.

#### 3.6.2. Further Outcome Measures

Other outcomes include nationality, age, sex, federal state, place of residence, and region (urban vs. rural); height, body weight, and calculated BMI; marital status; highest academic qualification; current health status; professional role as (a) student during study progress (current semester) and highest graduation, field of study, pedagogical/didactical or subject-specific orientation, and (b) lecturer/academic staff during employment and extent (full/part-time), type of college/university, and field of activity; current physical exercise behavior including motives, sports type, duration, intensity, and frequency, race participation, and sports club member; current adherence to diet type including motivation and duration, availability at buffet/canteen, fluid intake/day, fruit and vegetable intake (portions and frequency), intake of specific foods/food groups, daily meals, and self-assessed macronutrient distribution; leisure time activities; individually assessed wellness and health promoting lifestyle behaviors including (i) PA, sports & exercise (type, frequency, duration, and intensity), (ii) food (animal, plant, and supplements), (iii) sleep (duration and quality on work days and weekends), and (iv) other lifestyle factors combined/individually (e.g., amount and frequency of alcohol intake and smoking); (v) hypersensitivity reactions considering food/ingredient allergies/intolerances, chronic/serious illness, and quality of life; and (vi) independent data on lifestyle and behavioral changes during the COVID-19 pandemic.

Data will be analyzed in differentiated categories based on diet type and sociodemographic variables. Potential associations between variables at different study levels will be examined according to study questions and subsequent hypotheses. Specific study biases will be considered while interpretations and potential limitations will be notified in final reports. Findings will be reported through several scientific papers during the upcoming years.

### 3.7. Ethical Principles

The present study was conducted per the medical professional Codex, the Declaration of Helsinki 1996, good clinical practice guidelines, and Data Security Laws. Study participation was voluntary and could be terminated at any time without providing a reason or negative consequences.

#### 3.7.1. Written Informed Declaration of Consent and Publication

Prior to filling in the online questionnaire, and in addition to the information provided at the official website (https://uni.science2.school/en, accessed on 16 November 2022), study participants were provided with written information about the content, aim, and procedure of the study (participation was voluntary; data collection was anonymous and confidential; and data were processed exclusively for scientific purposes) and were asked to give their written consent to participate in the survey regarding the subsequent publication of the study results. If the respondent discontinues participation in the study, the respective data records will be deleted.

#### 3.7.2. Vote of the Ethics Board of the main Tertiary Educational Entities

This Austria nationwide study is supported by the Federal Ministry of Education, Science, and Research (BMBWF) Department 1/7—School and University Sports. The study was approved by the ethics board of the main tertiary educational entity, the Rectorate of the University College of Teacher Education Tyrol (PHT-HSa-17-Z1.8–5n_4927; 22 March 2021) and supported by the ethics board of the university, and the Vice rectorate for Research of the Leopold-Franzens University of Innsbruck (Certificate of good standing, 22/2021; 6 April 2021), which was of crucial importance when contacting the colleges/universities. The final step to get permission to start the study and conduct the survey in the tertiary educational setting in Austria (nationwide) was the approval by the respective board of deans at 102 colleges/universities all over Austria. Due to the requests of both the respective lead entities and the respective college/university management, no further ethical vote (e.g., institutional review board or local ethical committee) was required for this study.

#### 3.7.3. Duties on Part of the Investigators

The research team is obliged to conduct the study in accordance with the present protocol and report and document amendments to the authorities in charge. The data will be used and evaluated exclusively within the framework of the present study.

#### 3.7.4. Assessment of the Benefit–Risk Ratio

Participation in this study was voluntary, without any additional risk for participants, and was sensible and justifiable from a didactic–pedagogical viewpoint in terms of the Austrian tertiary educational context. Participants did not receive any financial compensation for participating in the study.

### 3.8. Data Security

All data will be treated in accordance with the applicable data protection regulations and data security laws. The online survey, including the databases and the website, was hosted on a secure server of ITEG Engineers GmbH (ISO 27,001 certified; IT specialists and partners of the project). The online survey was hosted on a dedicated virtual server and was https only, i.e., all relevant data was transferred SSL-encrypted. Access to the data (server as well as file and database backup) is limited only to the IT staff of the study team and the principal investigator. Security measures include a local firewall on the server and regular security updates of the operating system and applications. The SSH access is limited to SSH key authentication; the FTP access or other unencrypted access is blocked on the server side. The IP addresses of aggressively attacking clients are automatically blocked temporarily. The data were collected and stored anonymously. No IP addresses of the participants were stored in the survey or database. Therefore, the assignment of IP addresses or any personal information of participants of the survey was not possible, and no further step towards anonymization was necessary.

### 3.9. Power Analysis, Calculation of Case Number Scenarios and Representativity

The findings of the present study will be presented considering the STROBE and/or CONSORT criteria (https://www.strobe-statement.org/?id=available-checklists, http://www.consort-statement.org/, accessed on 16 November 2022).

Mainly age (18 ≥ 35 ≥ 65 years) and sex (male, female) were used for estimating sufficient case numbers for study groups. The data will be representative of the two age groups and sex on a 95% confidence interval (95%-CI) level (with a margin of error determined accordingly: ME = 0.025). Table 1 shows calculations of case number scenarios for different associations based on diet type.

Explanation for Scenario of both the Subsamples:

(i) Students. For the estimation of a proportion of 15.1% (two sided 95%-CI) ± 2.5% accuracy, a sample size of n = 788 is required after data clearance;
(ii) Lecturers/Academic Staff. For the estimation of a proportion of 9.45% (two sided 95%-CI) ± 2.5% accuracy, a sample size of n = 526 is required after data clearance.

The CI scenarios for proportions were performed using normal approximation (n large). The Software R (version 4.1.3; 10 March 2022) was used for the case number estimation.

Comparison between groups will be made using the appropriate chi-square contingence test with a level of significance of 0.05 and a power of 0.8 (effect size of students: w = 0.12; effect size of lecturers/academic staff: w = 0.13). For statistically reliable and representative results of any one particular variable, a sample size of n = 573 (lecturers/academic staff) and n= 694 (students) was needed in order to detect a 4% (lecturer) and a 7% (student) difference between dietary subgroups (mixed vs. vegetarian vs. vegan) and to reach 80% power with a two-sided test (95%-CI) with an alpha value of 0.05. Table 2 displays the associated estimations by the power analysis performed.

Explanation for Students Data Cube: for linking two or three variables (strata), a respective sample size of n = 1363 and n = 1834 was necessary for dietary subgroup comparison in students (e.g., age 18–35 years * sex male; or age 18–35 years * sex male * field of study) with the estimated effect size w = 0.10.

### 3.10. Statistical Methods and Data Analysis

Stratified subgroup analyses, including descriptive reports, will be conducted on a descriptive level. The statistical analyses will be performed using the latest version of statistical software according to the required statistical methods corresponding to the available data sets. All results will be presented as arithmetic means and standard deviations (SD) for metric variables or median and interquartile range (IQR) as absolute and relative values for categorical data. To display the graphical presentations, different types of charts and plots will be used. Multivariate regression analyses will be used to calculate the effects of kind of diet, age, sex, BMI, activity level, etc. ANOVA (analysis of variance) will be performed to identify individual differences in sex, age, BMI, etc., as well as years of adherence to the particular kind of diet and experience in sports & exercise, motivation for a specific kind of diet and sports & exercise; kind of sport and discipline (social form, informal/formal, and indoor/outdoor); and participation in events/competitions, sports club membership, additional sports, etc. The level of statistical significance was set at *p* ≤ 0.05.

## 4. Discussion and Future Perspectives

Evidence-based knowledge is the basis for nations and global operating health organizations to set their policies, guidelines, and recommendations. In the area of public health, understanding the interaction between the components of lifestyle is a fundamental concern of many health experts. This has originated from the great importance of lifestyle in improving health status of susceptible populations (e.g., young adults), especially as an intervention to treat chronic diseases [1,11,13]. The present Austria-nationwide study follows a novel approach to survey the lifestyle patterns of tertiary-level students and lecturers/academic staff by focusing on the prevalence of vegan, vegetarian, and mixed diets linked to PA, sports & exercise patterns.

### 4.1. Dual Approach of Health

The dual approach of sustainable health has been shown to have a powerful impact on overall health and wellbeing. It is well-known that diet and physical activity can independently enhance the effectiveness of each other [18,21,128], leading to lifelong health [27,28] as well as controlling the prevalence of overweight/obesity [18,25,27,29]. Research shows that health and body weight management approaches that incorporate a combination of physical exercise and dietary strategies are likely to yield better outcomes than single-component strategies [24,25,26,27]. However, due to the complex interaction between these components [20], it is not straightforward to determine the effects of each component independently. From an economic and social perspective, this approach is also considered a highly effective tool for reducing the monetary burden at both individual and public health levels [129,130,131]. Compared with the conventional treatment methods (e.g., medication and surgeries), adhering to a healthy diet (particularly whole food plant-based) combined with regular PA, sports & exercise (especially at a moderate intensity) not only provides a wide variety of direct and indirect health benefits, but also represents a low-cost and low-risk lifestyle intervention to manage a large number of diseases [32,34,132,133,134,135].

### 4.2. Diet Type

Health benefits of plant-based diets, especially in the management of certain chronic disease [33], have been emphasized by the Academy of Nutrition and Dietetics [111]. A significant reduction (up to 34%) in all-cause mortality has also been reported following a plant-based diet compared with a mixed diet [136,137,138]. In addition, people who follow vegan or vegetarian diets are reported to typically have a healthier BMI than omnivores [139,140,141]. This outcome may be due to the characteristics of plant-based foods (e.g., low-calorie alongside high density of micronutrients and dietary fiber) compared with animal-based foods (e.g., high calorie and high fat, protein, and sodium) [111,142,143] implying their importance in sustaining healthy body weight. Regardless of such nutritional differences, data have shown that vegan and vegetarian people may have further healthy lifestyle habits, including a higher involvement in sports & exercise activities [98,144] as well as a lower alcohol intake [98,99,145]. However, it should be considered that diet type, per se, may not be the ultimate indicator of a healthy nutritional pattern, since even vegans may have unhealthy nutritional habits (e.g., overconsumption of processed foods, sugar, salt, and refined carbohydrates), especially if the diet is planned inappropriately [32,102,146]. In general, assuring adequate consumption of all essential nutrients has been emphasized for preventing the risk of nutritional insufficiencies and decreasing the likelihood of most chronic diseases [147]. Hence, adequate consumption of fruits and vegetables with a balanced fluid intake and avoiding unhealthy food/ingredients (e.g., sugar, salt, and refined and processed foods) are considered key elements of a healthy diet irrespective of the diet type [148,149,150].

In some countries, such as Portugal [151], there is a mandatory law to offer at least one vegan dish in public entities (including schools, universities, hospitals, prisons, etc.). Consistently, the American Medical Association (2017) recommends that hospitals and medical facilities not only offer/serve plant-based meals and vegan options to improve the health of their patients, staff, and visitors, but at the same time eliminate animal products from their menus [152,153].

### 4.3. The COVID-19 Pandemic and Health-Related Behaviors

Opportunities to follow health behaviors may be partially influenced by limiting factors that are beyond the individual’s control and intention. For example, the current COVID-19 pandemic and the associated social problems (including social lockdowns) have considerably limited healthy behaviors such as PA opportunities [92,154]. It has been reported that the diminished access to fresh foods during this pandemic has resulted in an increased consumption of highly processed foods, which are typically high in salt, sugar, and saturated fat [155]. An Austrian study showed that during the COVID-19 pandemic, about half of Austrian adults have successfully maintained their health-oriented lifestyles (54%) or healthy diets (47%), with significant sex- and age-specific differences (the predominance of females over males and young over older generations) [154]. In this regard, two-thirds of Austrian young people and adults (over 14 years old) were reported to be physically active at least once a week [53]. According to the WHO, regular engagement in physical exercise at home combined with a healthy diet can strengthen the immune system and reduce the likelihood of viral diseases including SARS-CoV [156,157]. In addition, involvement in PA, sports & exercise is considered an effective strategy to reduce psychological distress during social lockdowns [157]. From a social and public health perspective, and given the fact that the current COVID-19 pandemic is more than a medical problem, the need for a special focus and a fundamental re-evaluation of healthy lifestyle behaviors as key factors in health care appears to be of urgent concern for national and global health.

### 4.4. Education and Health

Education (especially higher education) plays a critical role in improving and maintaining healthy lifestyles. Poor health behaviors are more prevalent in socially disadvantaged populations with limited access to didactic programs than in those with higher educational levels [73,74,75]. A large-scale study investigating the association between the level of education and health-related behaviors, including sport, exercise, and diet, showed significant differences between primary and higher education levels in health behaviors among adults [158]. Consistently, results from an Austrian investigation confirmed that a higher level of education is significantly associated with higher health literacy, with two-thirds of Austrian adults having adequate or very good health literacy [159]. However, 93.2% of Austrian adults aged 25 and older were reported to die from NCDs in 2019 [160]. Recently, an umbrella review identified education as a highly effective public health exposure for improving health and reducing morbidity as well as mortality from infectious diseases [161], indicating the key potentials of educational settings (primary up to tertiary level) to control NCDs and their risk factors. Due to increased social health problems in adolescence and young adulthood during the past decades, the health of younger generations is becoming the focus of educational policy and pedagogical measures. This result is in line with the well-established fact that health behaviors develop and shape during adolescence and emerging adulthood and track over time into older ages, thus becoming increasingly difficult to change in matured adulthood and later life stages [56,67,162,163]. In this regard, medical schools and teacher education centers need to implement healthy lifestyle courses and training in their educational curricula to promote health-related skills and qualifications of future teachers and physicians who are role models and multipliers for pupils, students, and patients.

### 4.5. Health at College/University Setting

College and university students are in a critical period of life, coined as emerging adulthood (18–25 years), for adopting and stabilizing sustainable and lifelong healthy behaviors [60,61,67,164]. Due to the increased prevalence of unhealthy lifestyle patterns as well as the higher rate of health-related risk factors (e.g., excess body weight, psychological distress, and poor dietary habits) among the current generation of university students [60,61,62,63], this cohort needs specific attention. Evidence shows that social environment (e.g., educational settings) is considered a critical contributor to the establishment of health behaviors in university students [65]. In addition to the age-related characteristics (i.e., at emerging adulthood), university life is independently associated with psychological stressors that undesirably affect health and future life [66]. Altogether, serious consideration must be applied in health-related educational policies to enhance health literacy and, consequently, strengthen the opportunities for sustainable individual and professional health behaviors from early adulthood (student) into matured adulthood (lecturers/academic staff) and the old/senior age.

Interspersed in the background of the global health crisis and its underlying data, it appears that educational settings (particularly the staff; e.g., teachers and lecturers) lack adequate health knowledge and/or the qualifications for translating and transferring competence-orientated health literacy to pupils and students (i.e., future generations). The health of principals and staff at all educational levels is not only a prerequisite for a high-quality basic, general, and specific education, but crucial for improving public health due to the direct and indirect impacts on student lifestyle choices [165,166] since they make up a considerable portion of society. It has been reported that about 2% of the European working population (~5 million people) are teachers and educational staff working at different educational levels [167]. This population is a characterized occupational group with various roles as educators, partners, role models, counselors, supervisors/mentors, social directors, professional managers, and political theorists [168]. Therefore, the existing concern for the health of educational and academic staff seems not to be an individual “private matter” per se but a contribution to the public education system [99,169].

### 4.6. Practical Implications

Results from the present study, especially together with the findings of the recently conducted school study, *From Science 2 School: Sustainably healthy–active & veggie*, will potentially provide a sound basis for reflecting on current health promotion measures (particularly the dual approach of health) based on evidence-based results on health behaviors, which will ultimately help to reflect, evaluate, and update the current health-related recommendations. In addition, this study can make a significant contribution to development of an overview of the current social trends of health behaviors, especially in the respective peer groups at tertiary educational settings. As a result of a sustainable healthy upbringing, a healthy transition from childhood to adulthood occurs, and therefore, healthy future parents and populations will be grown to make healthier societies and nations [170].

The findings of this study will provide a meaningful contribution by adding novel evidence to the body of science with a special focus on health behaviors, especially on the permanent linkage of sports & exercise engagement to diet types in the context of tertiary education. In addition, the present study will provide information about the association between the COVID-19 pandemic and lifestyle behaviors considering the diet and sports dual approach to sustainable health and several sociodemographic categories. The results are expected to help empower colleges and universities to design future offerings that consistently combine healthy and sustainable choices of ingredients, foods, meals, and dishes across their environments (e.g., vending machines, canteen, and catering) with regular opportunities of sports & exercise activities (e.g., cross course and cross curricular, interdisciplinary events across departments, and tertiary educational entities, etc.). Therefore, the findings will potentially aid in:

1. The justification of the fundamental dual approach of “Healthy Eating, Active Living” [22,23] as a minimum recommendation for sustainable health, which—based on sound evidence—is considered a highly effective, safe, and low-cost strategy to promote the health of students and lecturers/academic staff in order to tackle individual and public health issues, and ultimately help improve health status and prevent NCDs;
2. Encouraging policy and decision makers (e.g., federal authorities, stakeholders, board of deans, educational multipliers, and even families) to reflect, evaluate, and update the current health promotion offers, and to implement the dual approach to health in everyday scenarios and situations at tertiary educational settings and environments (e.g., curricula, sports & exercise opportunities, public catering of buffets, canteens, and cafeterias);
3. Developing evidence-based and up-to-date health-related knowledge, skills, and competencies required for a sustainable health-orientated action readiness (regardless of socioeconomic background) through competence-oriented teaching to help achieve better individual health and develop professional health-related qualifications, particularly in emerging adulthood.

As the seamless follow-up to the recently conducted school study (www.science2.school/en, accessed on 16 November 2022), the present S2HU study has been developed to transfer the questions of the secondary to the tertiary education level in order to continue the knowledge gain and generate consistent results tracking from childhood to emerging adulthood. Together with the results from the previous school study (i.e., S2S), the cumulative findings have the potential to provide a sound basis for reflection, evaluation, and application of current health promotion measures (specifically, the dual approach of health) on the current curricula. Furthermore, the results may serve as a basis to identify possible options for action and reconsideration of practical recommendations. Thus, the design of future physical activity and nutrition—at best dual—offers is possible, especially for the peer groups in tertiary education settings, such as health education and training, health literacy, and health-related educational research. Moreover, this study might be pragmatic to:

1. Transfer the results to the public by first addressing the college/university settings, students (any discipline; not only limited to future teachers and doctors), and lecturers/academic staff as education professionals and health multipliers;
2. Translate the findings into health-orientated actions and sustainable health-related action readiness (e.g., by recommending this safe, effective, and low-cost dual tool to policy and decision makers, multipliers, and experts in the health and education sector) by means of implementing this approach in tertiary teaching and training and everyday scenarios as a basic extension of the tertiary educational entity that is applicable for different populations (e.g., family, community, teachers, therapists, family/primary care and specialized physicians, dietitians, nutrition and sports experts, and educational supervisors/coaches);
3. Apply the results most effectively in everyday college and university scenarios through creating and implementing further tertiary curricula-based competence-orientated education and training offers and raising awareness of state-of-the-art knowledge, skills, and competencies in order to (i) empower the peer group of tertiary students, and (ii) establish the basic dual approach for sustainable health as a minimum recommendation for health promotion in tertiary curricula as an educative, teaching, and research goal in line with the state mandate, thus seamlessly closing the gap between secondary and tertiary educational levels. By deliberately promoting the qualifications required to manage long-term individual health throughout (updated) tertiary level curricula, the evolution of programs and modules with regard to compulsory introductory and basic courses leading to advanced and in-depth courses and materials, especially for, but not limited to, future teachers and doctors, lecturers/academic staff, will ultimately aid to improve the future public health of nations.

The present study is based on the Austrian state mandate at secondary level curricula [171,172,173,174,175], and therefore, follows the “prevention first” approach [4]. Consistently, the European Parliament has emphasized the “shift to prevention” as the most important approach to consider for interventions, measures, and agendas for improving public health [176]. However, the Austrian educational curricula [171,172,173,174,175,177] still have considerable potentials untapped to draw the attention of education experts and health specialists (i.e., the dual approach of health has yet to be thoroughly implemented). Therefore, the current gap remains unbridged regarding the translation and connection of scientific evidence to the practical settings to reach a more integrated and holistic lifestyle-focused approach, which appears to be the most promising strategy to stop and reverse the increasing prevalence of health problems in younger generations. This will also need sequencing and follow-up of curricular enrichment of health promotion topics from secondary up to tertiary level education (especially medical schools, pedagogy study/teacher education, and training at college/university) to update and standardize the associated modules/lectures. In perspective, results from the present study will help establish health-related knowledge, health-focused action competence, and sustainable action readiness through tertiary curricula implemented by education and training along with adequate offers and occasions concerning the individual health status of college and university students and their professional roles within their respective occupational areas (e.g., as future parents, teachers, lecturers/academic staff, therapists, nurses and physicians, and medical, health, sports and nutrition experts, policy and decision makers, or other multipliers and role models).

Considering the increasing prevalence of vegetarian/vegan diets [44,45,46,47,48] and the advanced importance of health behaviors in childhood, adolescence, and emerging adulthood [57,58,59,60,61], it is suggestible that plant-based options should be widely available in public catering of educational centers (e.g., buffets, canteens, refectories, and vending machines). In addition, the associated evidence-based knowledge of the consensus on both food as medicine and PA, sports & exercise as medicine should also be included in the curricula as well as teaching, learning, and training materials of schools, colleges, and universities [44,178,179]. Therefore, results from the present study will also be of help for policy and decision makers in the tertiary educational setting (including governmental and federal authorities, college/university management, education principals and lecturers/staff, and families) to evaluate, reflect, and update programs, materials, measures, and opportunities in everyday school, college, and university scenarios and educational environments and settings.

*Legal Action to Implement the Findings in the Educational Context.* Legislation and case law regarding plant-based diets are also applicable as the new field of tension in the school, college, and university settings, since according to the current trends, it is estimated that at least 2–5 students adhere to a vegan or vegetarian diet in each group of 25 students, as an average class. In the educational context of German and Austrian public catering, individual preferences and dislikes must be considered in the food offered as a result of psychosocial significances of diet [180]. Health promotion and education, preferably via the dual approach of sustainable health, is one of the top learning objectives of didactic interventions and educational principles of the Austrian education system. As declared by the UN SDGs, the WHO Voluntary Global Targets on NCDs, and the UNESCO Learning Objective “Cross-cutting Key Competencies”, health is an overarching objective for educational entities and policymakers to consolidate it into education and curricula [69,70,71]. Consistently, the WHO has also recommended that school and educational policies should support the adoption and maintenance of healthy lifestyle behaviors as a basis to improve public health [181]. Despite these facts and considering that health education is highly relevant to all compulsory subjects, current approaches have considered it primarily as an extra-curricular task, mainly under the umbrella of the compulsory subject “Physical Education” [171,172,173,174,175], which seems to be insufficient to cover all health-related topics. The interdisciplinary and complex knowledge on health as an educational mandate should be centered seamlessly in the tertiary education settings through teaching/training and research mandates, specifically established in the curricula of pedagogy studies and life sciences.

### 4.7. Limitations and Strengths

Due to the voluntarily nature of the participant recruitment, not all students and lecturers/academic staff at colleges and universities Austria nationwide were within reach of the chosen recruitment method. In addition, the cross-sectional design of the survey in which the data were generated based on self-assessment and self-report may result in socially desired statements such as over-reporting (e.g., longer duration of PA) and/or underreporting (e.g., lower body weight). However, to limit the likelihood of misreporting to a minimum and increase the reliability and validity of the data at the same time, several control questions were implemented in different parts of the survey to identify and control potential conflicting statements. Accordingly, conflicting data sets will be revised (shifted cases) or removed from the subsequent analyses within the data clearance process. Similar to any comparable study, confounding factors (including all direct and indirect lifestyle-related parameters) may potentially affect the findings and the associated interpretations. Based on specified research questions and (co-)variables, details about potential confounders will be addressed in the subsequent reports. The validation process for the questionnaire further showed that most of the items must be assessed individually, with the exception of the EMI-2 questionnaire [109,122,123]. This occurrence means that most variables in the study are based on a single-item construct leading to different limitations, i.e., lower precision in representing the related attribute, low number of discrimination points (meaning larger sample sizes are required), and impossibility to assess internal consistency [182]. Nonetheless, recent research has demonstrated that single-item questionnaires may be considered valid [183] and can be consistently used in the social sciences as they are easier to apply [184,185] and may significantly reduce the problems associated with lengthy surveys [186].

However, the present project includes some strengths. As the ongoing COVID-19 situation during the summer semester in 2021 was of urgent concern and highly relevant to the data collection, not only affecting the public but also tertiary educational entities, the study was even adapted to map COVID-19-related issues. Considering the limited information on current health behaviors in emerging adulthood, especially the lack of data on current lifestyle behaviors discriminated by diet type, and regarding the current COVID-19 situation at Austrian colleges and universities, it is necessary to expand the database with epidemiological results on health behaviors in the important life phase transitioning from childhood to adulthood, specifically emerging adulthood.

Since current scientific evidence on the main research question is limited to the overall social context, and since information is lacking in particular (1) on students vs. lecturers/academic staff and (2) on the tertiary educational context, i.e., college/university setting, the present study is the first to investigate the dual approach to sustainable health, i.e., current nutritional behavior combined with PA behavior, and the associated epidemiological as well as demographic–biometric aspects, conducted Austria nationwide, based on a large sample. Moreover, this study assesses the abovementioned issue within the bigger framework of a multidimensional approach by including other lifestyle factors by design, taking into account a large sample of target groups in tertiary education settings. This project aims to provide a large data set to help identify distinct differences between the various subgroups to allow for comparing different study groups and subgroups (students vs. lecturers/academic staff; omnivorous, vegetarian, and vegan; active vs. inactive; college vs. university; orientation of the tertiary educational institution; subject area; federal states; and place of residence), which also includes assessment of important demographic and anthropometric parameters (e.g., sex, age, and BMI) and other health-related aspects (health status, quality of life, etc.) with the main outcome variables to result in robust findings. With the sample size of 4510 students and 1043 lecturers/academic staff (at data closure, previous to data clearance), the effort for representative data seems reasonable to be achieved in both study populations in order to be able to reflect and transfer the results obtained concerning health benefits in the tertiary education sector (e.g., curricula, offers regarding exercise & sport, as well as buffet, canteen, and cafeteria). The representativeness can be determined regarding the factors such as demographic and anthropometric information as well as data on the educational context, current activity levels, dietary behaviors, and more.

## 5. Conclusions

Despite the importance of plant-based diets in health along with the growing number of vegans and vegetarians worldwide, there is a lack of data on health-related behaviors of those who follow sustainable plant-based vs. traditional animal-based diets, especially the susceptible populations of health behaviors (e.g., young adults). Taking a large sample into account (n = 5553), the present multidisciplinary study is the first to map the prevalence of omnivorous, vegetarian, and vegan diets linked to PA, sports, and exercise; it will provide an important contribution to the limited information about the association of diet type with different lifestyle behaviors but especially physical exercise patterns. The dual approach of sustainable health (i.e., adherence to a healthy diet combined with regular physical exercise) offers a promising “prevention first” perspective for sustainable and lifelong health. While the present findings will be analyzed based on differentiated sociodemographic categories, the influence of the COVID-19 pandemic on health-related behaviors will also be evaluated. In addition to this study, future interventions should be designed and conducted to provide comparable data for a deeper understanding of the integrated role of diet type and PA, sports, and exercise on health behaviors along with other lifestyle variables among different populations.

## Figures and Tables

**Table 1:** Calculated case number scenarios for study groups.

	Mensink et al. (2016) [47] German Norm Population (18–69 Years)	Wirnitzer et al. (2021) [98] Austrian Secondary Level Pupils (10–19 Years)	Wirnitzer et al. (2022) [99] Austrian Secondary Teachers/Principals (20–65 Years)
Confidence level	95%	95%	95%
Alternative Hypothesis	2-sided interval	2-sided interval	2-sided interval
Expected proportion (%)	6.5%	15.1%	9.45%
Margin of Error (ME; distance from proportion to limit)	2.5%	2.5%	2.5%
Power (accepted min. 80%)	96% (0.96)	91% (0.91)	100% (0.1)
Number per cell per stratum	374	788	526

**Table 2:** Power calculations for chi-square tests regarding diet linked to relevant variables (strata).

	df (n)	Effect Size (w)	Level of Significance	Power	N (Total)
Students [diet]	2	0.12	0.05	0.8	694
[diet] * [age * sex]	6	0.10	0.05	0.8	1363
[diet] * [age * sex * field of study]	14	0.10	0.05	0.8	1834
Lecturers/Academic Staff [diet]	2	0.13	0.05	0.8	573

Note. The basis scenarios for students and lecturers/academic staff regarding “diet” are based on the most relevant scientific literature. As there is no literature available for the refined student scenarios, the effect size (w) was set to a smaller value (0.10).

## Data Availability

Not applicable.

## Supplementary Materials

The following supporting information can be downloaded at: https://www.mdpi.com/article/10.3390/ijerph192215313/s1, Questionnaire for students and lecturers/academic staff at Austrian colleges and universities of tertiary educational level (in German).
