# Supplementary material for: Study Protocol of “Sustainably Healthy—From Science 2 Highschool & University”—Prevalence of Mixed, Vegetarian, and Vegan Diets Linked to Sports & Exercise among Austrian Tertiary Students and Lecturers/Academic Staff
Source: Int J Environ Res Public Health. 2022 Nov 19;19(22):15313. doi: 10.3390/ijerph192215313 (PMC9690980; doi:10.3390/ijerph192215313)
Supplement: Supplementary file 1 [file ijerph-19-15313-s001.zip › ijerph-2014944-supplementary.pdf]

Nachhaltig gesund – From Science 2 Highschool & University

Herzlich willkommen bei der Online-Umfrage zur Hochschul- & Universitäts-Studie

‚Nachhaltig gesund – From Science 2 Highschool & University‘!

In dieser Umfrage sind 127 Fragen enthalten.

TEIL A – ZUR PERSON

Die Fragen beziehen sich NICHT ausschließlich bzw. sind NICHT limitiert auf den COVID-19 Lockdown (03-05/2020 bzw. 11/2020-02/2021).

1. Ihre Nationalität? *

Bitte wählen Sie nur eine der folgenden Antworten aus:

- ◯ Österreich
- ◯ Andere

1.1 Andere Nationalität: *

Beantworten Sie diese Frage nur, wenn folgende Bedingungen erfüllt sind:
Antwort war 'Andere' bei Frage '1 [Nationalitaet1]' (1. Ihre Nationalität?)

Bitte wählen Sie nur eine der folgenden Antworten aus:

- ◯ Afghanistan
- ◯ Ägypten
- ◯ Albanien
- ◯ Algerien
- ◯ Andorra
- ◯ Angola
- ◯ Antigua und Barbuda
- ◯ Äquatorialguinea
- ◯ Argentinien
- ◯ Armenien
- ◯ Aserbaidschan
- ◯ Äthiopien
- ◯ Australien
- ◯ Bahamas
- ◯ Bahrain
- ◯ Bangladesch
- ◯ Barbados
- ◯ Belgien
- ◯ Belize
- ◯ Benin
- ◯ Bhutan
- ◯ Bolivien
- ◯ Bosnien und Herzegowina
- ◯ Botsuana
- ◯ Brasilien
- ◯ Brunei Darussalam
- ◯ Bulgarien
- ◯ Burkina Faso

- Burundi
- Chile
- China
- Costa Rica
- Côte d'Ivoire
- Dänemark
- Demokratische Republik Kongo
- Deutschland
- Dominica
- Dominikanische Republik
- Dschibuti
- Ecuador
- El Salvador
- Eritrea
- Estland
- Eswatini
- Fidschi
- Finnland
- Föderierte Staaten Mikronesien
- Frankreich
- Gabun
- Gambia
- Georgien
- Ghana
- Grenada
- Griechenland
- Guatemala
- Guinea
- Guinea-Bissau
- Guyana
- Haiti
- Honduras
- Indien

- Indonesien
- Irak
- Iran
- Irland
- Island
- Israel
- Italien
- Jamaika
- Japan
- Jemen
- Jordanien
- Kambodscha
- Kamerun
- Kanada
- Kap Verde
- Kasachstan
- Katar
- Kenia
- Kirgisistan
- Kiribati
- Kolumbien
- Komoren
- Demokratische Volksrepublik Korea
- Republik Korea
- Kosovo
- Kroatien
- Kuba
- Kuwait
- Laos
- Lesotho
- Lettland
- Libanon
- Liberia

- ◯ Libyen
- ◯ Liechtenstein
- ◯ Litauen
- ◯ Luxemburg
- ◯ Madagaskar
- ◯ Malawi
- ◯ Malaysia
- ◯ Malediven
- ◯ Mali
- ◯ Malta
- ◯ Marokko
- ◯ Marshallinsel
- ◯ Mauretanien
- ◯ Mauritius
- ◯ Mazedonien
- ◯ Mexiko
- ◯ Moldau
- ◯ Monaco
- ◯ Mongolei
- ◯ Montenegro
- ◯ Mosambik
- ◯ Myanmar
- ◯ Namibia
- ◯ Nauru
- ◯ Nepal
- ◯ Neuseeland
- ◯ Nicaragua
- ◯ Niederlande
- ◯ Niger
- ◯ Nigeria
- ◯ Norwegen
- ◯ Oman
- ◯ Osttimor

- ◯ Pakistan
- ◯ Palau
- ◯ Panama
- ◯ Papua-Neuguinea
- ◯ Paraguay
- ◯ Peru
- ◯ Philippinen
- ◯ Polen
- ◯ Portugal
- ◯ Republik Kongo
- ◯ Ruanda
- ◯ Rumänien
- ◯ Russische Föderation
- ◯ Saint Kitts und Nevis
- ◯ Saint Lucia
- ◯ Salomonen
- ◯ Sambia
- ◯ Samoa
- ◯ San Marino
- ◯ São Tomé und Príncipe
- ◯ Saudi-Arabien
- ◯ Schweden
- ◯ Schweiz
- ◯ Senegal
- ◯ Serbien
- ◯ Seychellen
- ◯ Sierra Leone
- ◯ Simbabwe
- ◯ Singapur
- ◯ Slowakei
- ◯ Slowenien
- ◯ Somalia
- ◯ Spanien

- Sri Lanka
- St. Vincent und die Grenadinen
- Südafrika
- Sudan
- Südsudan
- Suriname
- Syrien
- Tadschikistan
- Tansania
- Thailand
- Togo
- Tonga
- Trinidad und Tobago
- Tschad
- Tschechische Republik
- Tunesien
- Türkei
- Turkmenistan
- Tuvalu
- Uganda
- Ukraine
- Ungarn
- Uruguay
- Usbekistan
- Vanuatu
- Vatikanstaat
- Venezuela
- Vereinigte Arabische Emirate
- Vereinigte Staaten von Amerika
- Vereinigtes Königreich
- Vietnam
- Weißrussland
- Zentralafrikanische Republik

- ◯ Zypern

2. Ihr Wohnort? *

Bitte wählen Sie nur eine der folgenden Antworten aus:

- ◯ Stadt
- ◯ Marktgemeinde
- ◯ Gemeinde

3. Ihr Bundesland in dem Sie studieren/arbeiten bzw. Standort Ihrer Universität/Hochschule? *

Bitte wählen Sie nur eine der folgenden Antworten aus:

- ◯ Burgenland
- ◯ Kärnten
- ◯ Niederösterreich
- ◯ Oberösterreich
- ◯ Salzburg
- ◯ Steiermark
- ◯ Tirol
- ◯ Vorarlberg
- ◯ Wien

4. Ihr Geschlecht? *

Bitte wählen Sie nur eine der folgenden Antworten aus:

- ◯ Männlich
- ◯ Weiblich
- ◯ Divers

5. Ihr Körpergewicht?

Wichtig!
Anmerkung 1: Ihr Körpergewicht OHNE Kleidung und Schuhe!
Anmerkung 2: Falls Sie schwanger sind, geben Sie bitte an, welches Gewicht Sie VOR Ihrer Schwangerschaft hatten!

Beispiel:
60,3 kg oder 77,8 kg
51 kg oder 85,0 kg *

❗ Ihre Antwort muss zwischen 20 und 300 liegen.

Bitte geben Sie Ihre Antwort hier ein:

[ ]

kg

6. Ihre Körpergröße?

Wichtig!
Anmerkung: Ihre Körpergröße OHNE Schuhe! *

Bitte wählen Sie nur eine der folgenden Antworten aus:

- ◯ 1,00
- ◯ 1,01
- ◯ 1,02
- ◯ 1,03
- ◯ 1,04
- ◯ 1,05
- ◯ 1,06
- ◯ 1,07
- ◯ 1,08
- ◯ 1,09
- ◯ 1,10
- ◯ 1,11
- ◯ 1,12
- ◯ 1,13
- ◯ 1,14
- ◯ 1,15
- ◯ 1,16
- ◯ 1,17
- ◯ 1,18
- ◯ 1,19
- ◯ 1,20
- ◯ 1,21
- ◯ 1,22
- ◯ 1,23
- ◯ 1,24
- ◯ 1,25
- ◯ 1,26

- 1,27
- 1,28
- 1,29
- 1,30
- 1,31
- 1,32
- 1,33
- 1,34
- 1,35
- 1,36
- 1,37
- 1,38
- 1,39
- 1,40
- 1,41
- 1,42
- 1,43
- 1,44
- 1,45
- 1,46
- 1,47
- 1,48
- 1,49
- 1,50
- 1,51
- 1,52
- 1,53
- 1,54
- 1,55
- 1,56
- 1,57
- 1,58
- 1,59

- ◯ 1,60
- ◯ 1,61
- ◯ 1,62
- ◯ 1,63
- ◯ 1,64
- ◯ 1,65
- ◯ 1,66
- ◯ 1,67
- ◯ 1,68
- ◯ 1,69
- ◯ 1,70
- ◯ 1,71
- ◯ 1,72
- ◯ 1,73
- ◯ 1,74
- ◯ 1,75
- ◯ 1,76
- ◯ 1,77
- ◯ 1,78
- ◯ 1,79
- ◯ 1,80
- ◯ 1,81
- ◯ 1,82
- ◯ 1,83
- ◯ 1,84
- ◯ 1,85
- ◯ 1,86
- ◯ 1,87
- ◯ 1,88
- ◯ 1,89
- ◯ 1,90
- ◯ 1,91
- ◯ 1,92

- 1,93
- 1,94
- 1,95
- 1,96
- 1,97
- 1,98
- 1,99
- 2,00
- 2,01
- 2,02
- 2,03
- 2,04
- 2,05
- 2,06
- 2,07
- 2,08
- 2,09
- 2,10
- 2,11
- 2,12
- 2,13
- 2,14
- 2,15
- 2,16
- 2,17
- 2,18
- 2,19
- 2,20
- 2,21
- 2,22
- 2,23
- 2,24
- 2,25

- 2,26
- 2,27
- 2,28
- 2,29
- 2,30
- 2,31
- 2,32
- 2,33
- 2,34
- 2,35
- 2,36
- 2,37
- 2,38
- 2,39
- 2,40

7. Ihr Alter? *

Bitte wählen Sie nur eine der folgenden Antworten aus:

- ◯ 16
- ◯ 17
- ◯ 18
- ◯ 19
- ◯ 20
- ◯ 21
- ◯ 22
- ◯ 23
- ◯ 24
- ◯ 25
- ◯ 26
- ◯ 27
- ◯ 28
- ◯ 29
- ◯ 30
- ◯ 31
- ◯ 32
- ◯ 33
- ◯ 34
- ◯ 35
- ◯ 36
- ◯ 37
- ◯ 38
- ◯ 39
- ◯ 40
- ◯ 41
- ◯ 42
- ◯ 43
- ◯ 44
- ◯ 45

- ◯ 46
- ◯ 47
- ◯ 48
- ◯ 49
- ◯ 50
- ◯ 51
- ◯ 52
- ◯ 53
- ◯ 54
- ◯ 55
- ◯ 56
- ◯ 57
- ◯ 58
- ◯ 59
- ◯ 60
- ◯ 61
- ◯ 62
- ◯ 63
- ◯ 64
- ◯ 65
- ◯ 66
- ◯ 67
- ◯ 68
- ◯ 69
- ◯ 70
- ◯ 71
- ◯ 72
- ◯ 73
- ◯ 74
- ◯ 75
- ◯ 76
- ◯ 77
- ◯ 78

- ◯ 79
- ◯ 80
- ◯ 81
- ◯ 82
- ◯ 83
- ◯ 84
- ◯ 85
- ◯ 86
- ◯ 87
- ◯ 88
- ◯ 89
- ◯ 90
- ◯ 91
- ◯ 92
- ◯ 93
- ◯ 94
- ◯ 95
- ◯ 96
- ◯ 97
- ◯ 98
- ◯ 99
- ◯ 100
- ◯ 101
- ◯ 102
- ◯ 103
- ◯ 104
- ◯ 105
- ◯ 106
- ◯ 107
- ◯ 108
- ◯ 109
- ◯ 110
- ◯ 111

- 112
- 113
- 114
- 115
- 116
- 117
- 118
- 119
- 120

8. Was ist hier Ihr höchster Schulabschluß? *

Bitte wählen Sie nur eine der folgenden Antworten aus:

- Kein Abschluß
- Hauptschule/Mittelschule (Sekundarstufe 1)
- Mittlere Reife/AHS-Gymnasium (Sekundarstufe 1)
- Fachhochschulreife (Sekundarstufe 2 ohne Matura/Abitur)
- Matura/Abitur (Sekundarstufe 2 mit Matura/Abitur)
- Fachhochschule/Pädagogische Hochschule etc.
- Universität
- Postgraduiert (Dr. usw.)

9. Ihr Familienstand? *

Bitte wählen Sie nur eine der folgenden Antworten aus:

- ◯ Allein lebend
- ◯ Getrennt lebend
- ◯ Verheiratet
- ◯ Geschieden
- ◯ Mit Partner lebend
- ◯ Verwitwet

10. Ich bin … *

Bitte wählen Sie nur eine der folgenden Antworten aus:

- ◯ Studierende/r
- ◯ Lehrende/r

10.1.1 Ich studiere im ...

Wichtig!
Mehrfachnennung möglich. *

Beantworten Sie diese Frage nur, wenn folgende Bedingungen erfüllt sind:
Antwort war 'Studierende/r' bei Frage '11 [DozStudi]' (10. Ich bin …)

Bitte wählen Sie alle zutreffenden Antworten aus:

- ☐ Bachelor-Studium
- ☐ Master-Studium
- ☐ Doktorats-/PhD-Studium

10.1.2a Bachelor-Studium im wievielten Semester? *

Beantworten Sie diese Frage nur, wenn folgende Bedingungen erfüllt sind:
Antwort war bei Frage '12 [IchStudiereIm]' (10.1.1 Ich studiere im ...Wichtig!Mehrfachnennung möglich.)

Bitte wählen Sie nur eine der folgenden Antworten aus:

- ◯ 1
- ◯ 2
- ◯ 3
- ◯ 4
- ◯ 5
- ◯ 6
- ◯ 7
- ◯ 8
- ◯ 9
- ◯ 10
- ◯ 11
- ◯ 12
- ◯ 13
- ◯ 14
- ◯ 15
- ◯ 16
- ◯ 17
- ◯ 18
- ◯ 19
- ◯ 20
- ◯ 21
- ◯ 22
- ◯ 23
- ◯ 24
- ◯ 25
- ◯ 26
- ◯ 27

- ◯ 28
- ◯ 29
- ◯ 30
- ◯ 31
- ◯ 32
- ◯ 33
- ◯ 34
- ◯ 35
- ◯ 36
- ◯ 37
- ◯ 38
- ◯ 39
- ◯ 40
- ◯ 41
- ◯ 42
- ◯ 43
- ◯ 44
- ◯ 45
- ◯ 46
- ◯ 47
- ◯ 48
- ◯ 49
- ◯ 50
- ◯ 51
- ◯ 52
- ◯ 53
- ◯ 54
- ◯ 55
- ◯ 56
- ◯ 57
- ◯ 58
- ◯ 59
- ◯ 60

- ◯ 61
- ◯ 62
- ◯ 63
- ◯ 64
- ◯ 65
- ◯ 66
- ◯ 67
- ◯ 68
- ◯ 69
- ◯ 70
- ◯ 71
- ◯ 72
- ◯ 73
- ◯ 74
- ◯ 75
- ◯ 76
- ◯ 77
- ◯ 78
- ◯ 79
- ◯ 80

10.1.2b Master-Studium im wievielten Semester? *

Beantworten Sie diese Frage nur, wenn folgende Bedingungen erfüllt sind:
Antwort war bei Frage '12 [IchStudiereIm]' (10.1.1 Ich studiere im ...Wichtig!Mehrfachnennung möglich.)

Bitte wählen Sie nur eine der folgenden Antworten aus:

- ◯ 1
- ◯ 2
- ◯ 3
- ◯ 4
- ◯ 5
- ◯ 6
- ◯ 7
- ◯ 8
- ◯ 9
- ◯ 10
- ◯ 11
- ◯ 12
- ◯ 13
- ◯ 14
- ◯ 15
- ◯ 16
- ◯ 17
- ◯ 18
- ◯ 19
- ◯ 20
- ◯ 21
- ◯ 22
- ◯ 23
- ◯ 24
- ◯ 25
- ◯ 26
- ◯ 27

- ◯ 28
- ◯ 29
- ◯ 30
- ◯ 31
- ◯ 32
- ◯ 33
- ◯ 34
- ◯ 35
- ◯ 36
- ◯ 37
- ◯ 38
- ◯ 39
- ◯ 40
- ◯ 41
- ◯ 42
- ◯ 43
- ◯ 44
- ◯ 45
- ◯ 46
- ◯ 47
- ◯ 48
- ◯ 49
- ◯ 50
- ◯ 51
- ◯ 52
- ◯ 53
- ◯ 54
- ◯ 55
- ◯ 56
- ◯ 57
- ◯ 58
- ◯ 59
- ◯ 60

- ◯ 61
- ◯ 62
- ◯ 63
- ◯ 64
- ◯ 65
- ◯ 66
- ◯ 67
- ◯ 68
- ◯ 69
- ◯ 70
- ◯ 71
- ◯ 72
- ◯ 73
- ◯ 74
- ◯ 75
- ◯ 76
- ◯ 77
- ◯ 78
- ◯ 79
- ◯ 80

10.1.2c Doktorats-/PhD-Studium im wievielten Semester? *

Beantworten Sie diese Frage nur, wenn folgende Bedingungen erfüllt sind:
Antwort war bei Frage '12 [IchStudiereIm]' (10.1.1 Ich studiere im ...Wichtig!Mehrfachnennung möglich.)

Bitte wählen Sie nur eine der folgenden Antworten aus:

- ◯ 1
- ◯ 2
- ◯ 3
- ◯ 4
- ◯ 5
- ◯ 6
- ◯ 7
- ◯ 8
- ◯ 9
- ◯ 10
- ◯ 11
- ◯ 12
- ◯ 13
- ◯ 14
- ◯ 15
- ◯ 16
- ◯ 17
- ◯ 18
- ◯ 19
- ◯ 20
- ◯ 21
- ◯ 22
- ◯ 23
- ◯ 24
- ◯ 25
- ◯ 26

- ◯ 27
- ◯ 28
- ◯ 29
- ◯ 30
- ◯ 31
- ◯ 32
- ◯ 33
- ◯ 34
- ◯ 35
- ◯ 36
- ◯ 37
- ◯ 38
- ◯ 39
- ◯ 40
- ◯ 41
- ◯ 42
- ◯ 43
- ◯ 44
- ◯ 45
- ◯ 46
- ◯ 47
- ◯ 48
- ◯ 49
- ◯ 50
- ◯ 51
- ◯ 52
- ◯ 53
- ◯ 54
- ◯ 55
- ◯ 56
- ◯ 57
- ◯ 58
- ◯ 59

- ○ 60
- ○ 61
- ○ 62
- ○ 63
- ○ 64
- ○ 65
- ○ 66
- ○ 67
- ○ 68
- ○ 69
- ○ 70
- ○ 71
- ○ 72
- ○ 73
- ○ 74
- ○ 75
- ○ 76
- ○ 77
- ○ 78
- ○ 79
- ○ 80

10.1.3 Ich studiere in der Studienrichtung ... und im Studienfach ...

Wichtig!
Mehrfachnennung möglich.

Beispiel:
Studienrichtung Lehramt – Studienfach Mathematik
Studienrichtung Fachwissenschaften – Studienfach Wirtschaftswissenschaften *

Beantworten Sie diese Frage nur, wenn folgende Bedingungen erfüllt sind:
Antwort war 'Studierende/r' bei Frage '11 [DozStudi]' (10. Ich bin …)

❗ Kommentieren wenn eine Antwort gewählt wird
Bitte wählen Sie die zutreffenden Punkte aus und schreiben Sie einen Kommentar dazu:

☐ Lehramt-1 (Hauptstudium):

☐ Lehramt-2:

☐ Lehramt-3:

☐ Fachwissenschaft(en)-1 (Hauptstudium):

☐ Fachwissenschaft(en)-2:

☐ Fachwissenschaft(en)-3:

10.2.1 Ihre Beschäftigung? *

Beantworten Sie diese Frage nur, wenn folgende Bedingungen erfüllt sind:
Antwort war 'Lehrende/r' bei Frage '11 [DozStudi]' (10. Ich bin …)

Bitte wählen Sie nur eine der folgenden Antworten aus:

- ◯ Bediensteter (Lehre & Forschung) an Universität bzw. Hochschule (Fach-HS, Pädagogische HS, privat, uvm.)
- ◯ Externer Lehrbeauftragter

10.2.2 Ihr Beschäftigungsausmaß? *

Beantworten Sie diese Frage nur, wenn folgende Bedingungen erfüllt sind:
Antwort war 'Lehrende/r' bei Frage '11 [DozStudi]' (10. Ich bin …)

❗ Wenn Sie 'Teilzeit in %:' auswählen, spezifizieren Sie bitte Ihre Auswahl im entsprechenden Textfeld.
❗ Nur Zahlen können in das 'Teilzeit in %:' begleitende Textfeld eingegeben werden.
Bitte wählen Sie nur eine der folgenden Antworten aus:

- ◯ 100 % (Vollzeit)
- ◯ Teilzeit in %: ______

10.2.3 An welcher Universität bzw. Hochschule (Fach-HS, Pädagogische HS, privat, uvm.) sind Sie hauptberuflich tätig? *

Beantworten Sie diese Frage nur, wenn folgende Bedingungen erfüllt sind:
Antwort war 'Lehrende/r' bei Frage '11 [DozStudi]' (10. Ich bin …)

❗ Wenn Sie 'Andere:' auswählen, spezifizieren Sie bitte Ihre Auswahl im entsprechenden Textfeld.
Bitte wählen Sie nur eine der folgenden Antworten aus:

- ◯ Universität
- ◯ Medizinische Universität
- ◯ Private Universität
- ◯ Pädagogische Hochschule
- ◯ Fachhochschule
- ◯ Andere: ____________

10.2.3a An welcher Universität bzw. Hochschule (Fach-HS, Pädagogische HS, privat, uvm.) sind Sie hauptberuflich tätig?

Beantworten Sie diese Frage nur, wenn folgende Bedingungen erfüllt sind:
Antwort war 'Universität' *oder* 'Medizinische Universität' *oder* 'Private Universität' *oder* 'Pädagogische Hochschule' *oder* 'Fachhochschule' bei Frage '19 [WelcherUni]' (10.2.3 An welcher Universität bzw. Hochschule (Fach-HS, Pädagogische HS, privat, uvm.) sind Sie hauptberuflich tätig?)

❗ Kommentieren wenn eine Antwort gewählt wird
Bitte wählen Sie die zutreffenden Punkte aus und schreiben Sie einen Kommentar dazu:

- ☐ Genaue Art/Bezeichnung

____

10.2.4 In welcher Fachrichtung (evtl. auch Disziplin) in Lehre und Forschung sind Sie tätig?

Beispiel:
Sportwissenschaft – Sportpädagogik
Physik – Experimentalphysik *

Beantworten Sie diese Frage nur, wenn folgende Bedingungen erfüllt sind:
Antwort war 'Lehrende/r' bei Frage '11 [DozStudi]' (10. Ich bin …)

Bitte geben Sie Ihre Antwort hier ein:

11. Wie würden Sie aktuell Ihren allgemeinen Gesundheitszustand einschätzen? *

Bitte wählen Sie nur eine der folgenden Antworten aus:

- ◯ Ausgezeichnet
- ◯ Sehr gut
- ◯ Gut
- ◯ Weniger gut
- ◯ Schlecht

TEIL B – BEWEGUNG & SPORT

Die Fragen beziehen sich NICHT ausschließlich bzw. sind NICHT limitiert auf den COVID-19 Lockdown (03-05/2020 bzw. 11/2020-02/2021).

Definition „Bewegung & Sport“
Bewegungs- und/oder Sport-Aktivität ... z. B. Wandern, Wassergymnastik, Yoga, oder Gartenarbeit uvm.

- *Mindestens 150 min/Woche moderate und/oder mindestens 75 min/Woche hohe Intensität, bei ...*
- *Erhöhtem Herzschlag/erhöhter Herzfrequenz, und*
- *Erhöhter Atmung/Atemfrequenz, und*
- *Erhöhter Körpertemperatur aufgrund von Bewegungs- und/oder Sport-Aktivität (vgl. nicht Sauna oder Solarium!), z. B. mir wir warm/heiß, ich schwitze, ich glühe etc.*

1. Betreiben Sie in Ihrer Freizeit Bewegung & Sport? *

Bitte wählen Sie nur eine der folgenden Antworten aus:

○ Ja

○ Nein

Warum betreiben Sie in Ihrer Freizeit keinen Sport bzw. sind Sie körperlich nicht aktiv? *

Beantworten Sie diese Frage nur, wenn folgende Bedingungen erfüllt sind:
Antwort war 'Nein' bei Frage '24 [BetreibenSport]' (1. Betreiben Sie in Ihrer Freizeit Bewegung & Sport?)

Bitte geben Sie Ihre Antwort hier ein:

2. Warum treiben Sie Sport bzw. sind Sie körperlich aktiv?

Ich treibe in meiner Freizeit Sport bzw. übe eine Bewegungsaktivität aus ... *

Beantworten Sie diese Frage nur, wenn folgende Bedingungen erfüllt sind:
Antwort war 'Ja' bei Frage '24 [BetreibenSport]' (1. Betreiben Sie in Ihrer Freizeit Bewegung & Sport?)

Bitte wählen Sie die zutreffende Antwort für jeden Punkt aus:

	**Ich stimme nicht zu 0**	**1**	**2**	**3**	**4**	**Ich stimme zu 5**
**... um schlank zu bleiben**	○	○	○	○	○	○
**... um mich gut zu fühlen**	○	○	○	○	○	○
**... damit ich besser aussehe**	○	○	○	○	○	○
**... um mir den Kopf freizumachen**	○	○	○	○	○	○
**... um meinen Körper gesund zu halten**	○	○	○	○	○	○
**... um Kraft aufzubauen**	○	○	○	○	○	○
**... weil ich mich gerne anstrenge**	○	○	○	○	○	○
**... um Zeit mit Freunden zu verbringen**	○	○	○	○	○	○

	Ich stimme nicht zu 0	1	2	3	4	Ich stimme zu 5
**... weil ich gerne versuche bei sportlichen Aktivitäten zu gewinnen**	○	○	○	○	○	○
**... um Gewicht abzunehmen**	○	○	○	○	○	○
**... weil es mich belebt**	○	○	○	○	○	○
**... um meinen Körper in Form zu halten**	○	○	○	○	○	○
**... weil mir Sport die Möglichkeit für schöne Bewegungen bietet**	○	○	○	○	○	○
**... weil es mir hilft, Spannungen abzubauen**	○	○	○	○	○	○
**... weil ich mich bei guter Gesundheit halten will**	○	○	○	○	○	○
**... um meine Ausdauer zu steigern**	○	○	○	○	○	○
**... weil ich Sport treiben befriedigend finde**	○	○	○	○	○	○
**... um den sozialen Aspekt des Sporttreibens zu genießen**	○	○	○	○	○	○
**... weil ich mich gerne messe**	○	○	○	○	○	○

	Ich stimme nicht zu 0	1	2	3	4	Ich stimme zu 5
... um mein Gewicht zu kontrollieren	◯	◯	◯	◯	◯	◯
... um meine Batterien wieder aufzuladen	◯	◯	◯	◯	◯	◯
... um meine Erscheinung zu verbessern	◯	◯	◯	◯	◯	◯
... um Stress zu bewältigen	◯	◯	◯	◯	◯	◯
... um mich gesünder zu fühlen	◯	◯	◯	◯	◯	◯
... um kräftiger zu werden	◯	◯	◯	◯	◯	◯
... wegen der Freude Sport zu treiben	◯	◯	◯	◯	◯	◯
... weil es mir Spaß macht mit anderen Menschen aktiv zu sein	◯	◯	◯	◯	◯	◯
... weil ich körperliche Wettkämpfe mag	◯	◯	◯	◯	◯	◯
... weil Bewegung mir hilft Kalorien abzubauen	◯	◯	◯	◯	◯	◯
... um attraktiver auszusehen	◯	◯	◯	◯	◯	◯
... weil es mir Freude bereitet die Schönheit menschlicher Bewegung im Sport zu erleben	◯	◯	◯	◯	◯	◯

	Ich stimme nicht zu<br>0	1	2	3	4	Ich stimme zu<br>5
... um Spannungen loszulassen	○	○	○	○	○	○
... um meine Muskeln aufzubauen	○	○	○	○	○	○
... weil ich mich am besten fühle, wenn ich Sport treibe	○	○	○	○	○	○
... um neue Freunde kennenzulernen	○	○	○	○	○	○
... weil ich finde, dass sportliche Aktivitäten besonders Spaß machen, wenn es sich um einen Wettkampf handelt	○	○	○	○	○	○
Andere Motive/Beweggründe	○	○	○	○	○	○

TEIL B – BEWEGUNG & SPORT

2a Welches sind Ihre anderen Motive/Beweggründe für Sport in der Freizeit? *

Beantworten Sie diese Frage nur, wenn folgende Bedingungen erfüllt sind:
Antwort war 'Ja' bei Frage '24 [BetreibenSport]' (1. Betreiben Sie in Ihrer Freizeit Bewegung & Sport?) *und* Antwort war '

4

' *oder* '

Ich stimme zu
5

' bei Frage '26 [WSport1]' (2. Warum treiben Sie Sport bzw. sind Sie körperlich aktiv?Ich treibe in meiner Freizeit Sport bzw. übe eine Bewegungsaktivität aus ... (Andere Motive/Beweggründe))

Bitte geben Sie Ihre Antwort hier ein:

2.1 Meine Top-3 Motive für Sport- bzw. Bewegungsaktivität in der Freizeit.

Wichtig! Bitte nehmen Sie eine Top-3-Reihung für Ihre 3 Haupt-Motive vor in Ihrer Freizeit sportlich bzw. körperlich aktiv zu sein – Reihung mit absteigender Bedeutung/Gewichtung Top 1-2-3: 1 für das wichtigste Motiv, 2 für das 2./zweit-wichtigste Motiv, 3 für das 3./dritt-wichtigste Motiv. *

Beantworten Sie diese Frage nur, wenn folgende Bedingungen erfüllt sind:
Antwort war 'Ja' bei Frage '24 [BetreibenSport]' (1. Betreiben Sie in Ihrer Freizeit Bewegung & Sport?)

❗ Bitte wählen Sie 3 Antworten aus.
Bitte nummerieren Sie jede Box in der Reihenfolge Ihrer Präferenz, beginnen mit 1 bis 37
Bitte wählen Sie mindestens 3 Einträge aus.
Bitte wähle Sie nicht mehr als 3 Einträge aus.

☐ ... um schlank zu bleiben

☐ ... um mich gut zu fühlen

☐ ... damit ich besser aussehe

☐ ... um mir den Kopf freizumachen

☐ ... um meinen Körper gesund zu halten

☐ ... um Kraft aufzubauen

☐ ... weil ich mich gerne anstrenge

☐ ... um Zeit mit Freunden zu verbringen

☐ ... weil ich gerne versuche bei sportlichen Aktivitäten zu gewinnen

☐ ... um Gewicht abzunehmen

... weil es mich belebt

... um meinen Körper in Form zu halten

... weil mir Sport die Möglichkeit für schöne Bewegungen bietet

... weil es mir hilft, Spannungen abzubauen

... weil ich mich bei guter Gesundheit halten will

... um meine Ausdauer zu steigern

... weil ich Sport treiben befriedigend finde

... um den sozialen Aspekt des Sporttreibens zu genießen

... weil ich mich gerne messe

... um mein Gewicht zu kontrollieren

... um meine Batterien wieder aufzuladen

... um meine Erscheinung zu verbessern

... um Stress zu bewältigen

... um mich gesünder zu fühlen

... um kräftiger zu werden

... wegen der Freude Sport zu treiben

... weil es mir Spaß macht mit anderen Menschen aktiv zu sein

... weil ich körperliche Wettkämpfe mag

... weil Bewegung mir hilft Kalorien abzubauen

... um attraktiver auszusehen

... weil es mir Freude bereitet die Schönheit menschlicher Bewegung im Sport zu erleben

... um Spannungen loszulassen

... um meine Muskeln aufzubauen

... weil ich mich am besten fühle, wenn ich Sport treibe

... um neue Freunde kennenzulernen

... weil ich finde, dass sportliche Aktivitäten besonders Spaß machen, wenn es sich um einen Wettkampf handelt

Ihre anderen Motive/Beweggründe

3.1 Welche Sommerssportart(en) betreiben Sie in Ihrer Freizeit?

Wichtig!
Mehrfachnennung möglich. *

Beantworten Sie diese Frage nur, wenn folgende Bedingungen erfüllt sind:
Antwort war 'Ja' bei Frage '24 [BetreibenSport]' (1. Betreiben Sie in Ihrer Freizeit Bewegung & Sport?)

Bitte wählen Sie alle zutreffenden Antworten aus:

- ☐ Schwimmen
- ☐ Laufen
- ☐ Radfahren
- ☐ Wandern
- ☐ Klettern
- ☐ Alpines Bergsteigen
- ☐ Mountainbiken
- ☐ Sonstiges Sommersport::

3.2 Welche Wintersportart(en) betreiben Sie in Ihrer Freizeit?

Wichtig!
Mehrfachnennung möglich. *

Beantworten Sie diese Frage nur, wenn folgende Bedingungen erfüllt sind:
((BetreibenSport.NAOK (/ls/index.php/admin/questions/sa/view/surveyid/159885/gid/2/qid/22) == "A1"))

Bitte wählen Sie alle zutreffenden Antworten aus:

- ☐ Skifahren
- ☐ Rodeln
- ☐ Langlaufen
- ☐ Skitourengehen
- ☐ Schneeschuhwandern
- ☐ Tanzen
- ☐ Ballsportarten
- ☐ Fitness-/Kraft-Training
- ☐ Sonstiges Wintersport::

3.3 Was ist Ihre Hauptsportart?

Wichtig!
Nur 1 Antwort zulässig. *

Beantworten Sie diese Frage nur, wenn folgende Bedingungen erfüllt sind:
Antwort war 'Ja' bei Frage '24 [BetreibenSport]' (1. Betreiben Sie in Ihrer Freizeit Bewegung & Sport?)

❗ Bitte wählen Sie eine Antwort
Bitte wählen Sie alle zutreffenden Antworten aus:

- ☐ {Sommersportarten_SQS01.question}
- ☐ {Sommersportarten_SQS02.question}
- ☐ {Sommersportarten_SQS03.question}
- ☐ {Sommersportarten_SQS04.question}
- ☐ {Sommersportarten_SQS05.question}
- ☐ {Sommersportarten_SQS06.question}
- ☐ {Sommersportarten_SQS07.question}
- ☐ {Sommersportarten_other}
- ☐ {Wintersportarten_SQW01.question}
- ☐ {Wintersportarten_SQW02.question}
- ☐ {Wintersportarten_SQW03.question}
- ☐ {Wintersportarten_SQW04.question}
- ☐ {Wintersportarten_SQW05.question}
- ☐ {Wintersportarten_SQW06.question}
- ☐ {Wintersportarten_SQW07.question}
- ☐ {Wintersportarten_SQW08.question}
- ☐ {Wintersportarten_other}

4. An wie vielen Tagen pro Woche betreiben Sie Sport in Ihrer Freizeit (Hobby, Training, und/oder Wettkampf)? *

Beantworten Sie diese Frage nur, wenn folgende Bedingungen erfüllt sind:
Antwort war 'Ja' bei Frage '24 [BetreibenSport]' (1. Betreiben Sie in Ihrer Freizeit Bewegung & Sport?)

Bitte wählen Sie nur eine der folgenden Antworten aus:

- ◯ 1 Tag/Woche
- ◯ 2 Tag/Woche
- ◯ 3 Tag/Woche
- ◯ 4 Tag/Woche
- ◯ 5 Tag/Woche
- ◯ 6 Tag/Woche
- ◯ Täglich (7 Tage/Woche)

5. Durchschnittliche Dauer Ihrer (täglichen) Sporteinheit?

Beispiel:
00:15 (=15 min)
01:00 (=1 h)
01:45 (=1 h 45 min)
02:30 (=2 h 30 min)
04:20 (=4 h 20 min) *

Beantworten Sie diese Frage nur, wenn folgende Bedingungen erfüllt sind:
Antwort war 'Ja' bei Frage '24 [BetreibenSport]' (1. Betreiben Sie in Ihrer Freizeit Bewegung & Sport?)

Bitte wählen Sie die zutreffende Antwort für jeden Punkt aus:

	**Stunden**	**Minuten**
	[illegible]	[illegible]
**ø Stunden/Sporteinheit**		

6. Nehmen Sie auch an sportlichen Wettkämpfen teil? *

Beantworten Sie diese Frage nur, wenn folgende Bedingungen erfüllt sind:
Antwort war 'Ja' bei Frage '24 [BetreibenSport]' (1. Betreiben Sie in Ihrer Freizeit Bewegung & Sport?)

Bitte wählen Sie nur eine der folgenden Antworten aus:

- Ja
- Nein

6.1 Ihr Ziel bei einem sportlichen Wettkampf? *

Beantworten Sie diese Frage nur, wenn folgende Bedingungen erfüllt sind:
Antwort war 'Ja' bei Frage '35 [TeilnSportWettk]' (6. Nehmen Sie auch an sportlichen Wettkämpfen teil?)

❗ Wenn Sie 'Anderes:' auswählen, spezifizieren Sie bitte Ihre Auswahl im entsprechenden Textfeld.
Bitte wählen Sie nur eine der folgenden Antworten aus:

◯ Durchkommen/Dabei sein ist alles!
◯ Freude und Spaß
◯ Mannschaft/Teamkollegen unterstützen
◯ Konkrete Wertung (z. B. Punkte, Zeit)
◯ Konkrete Platzierung (z. B. Sieg, Podium, Top 10)
◯ Anderes: ____________

7. Sind Sie aktives Mitglied in einem Sportverein? *

Beantworten Sie diese Frage nur, wenn folgende Bedingungen erfüllt sind:
Antwort war 'Ja' bei Frage '24 [BetreibenSport]' (1. Betreiben Sie in Ihrer Freizeit Bewegung & Sport?)

Bitte wählen Sie nur eine der folgenden Antworten aus:

◯ Ja
◯ Nein

TEIL C – ERNÄHRUNG

Die Fragen beziehen sich NICHT ausschließlich bzw. sind NICHT limitiert auf den COVID-19 Lockdown (03-05/2020 bzw. 11/2020-02/2021).

1. Wie ernähren Sie sich aktuell?

Definition Ernährungsformen in Anlehnung an die weltweit größte Fachgesellschaft für Ernährung, Academy of Nutrition and Dietetics (AND, 2015/2016):

Art der Ernährung	JA, diese Produkte esse ich:	NEIN, diese Produkte esse ich NICHT:
**Mischkost** (ich esse alles)	**„Alles"** z. B. Fleisch inkl. Wurst, Fleischkäse/Leberkäse, Schinken, Salami, Streichwurst, Käse, Butter, Milch, Milchprodukte, Fisch und sog. „Meeresfrüchte", Obst, Gemüse, Kartoffeln, Getreide, usw.	/
**Vegetarisch** (pflanzlich; lakto-ovo-vegetarisch)	**Milch und Milchprodukte, Eier und Eiprodukte** z. B. Käse, Butter, Joghurt, Topfen/Quark, Molke/Whey, usw.	**Fleisch, Fisch und sog. „Meeresfrüchte"** inkl. Wurst, Schinken, Salami, Fleischkäse/Leberkäse, Streichwurst, usw.
**Vegan** (rein pflanzlich)	**Pflanzliche Produkte** z. B. Obst, Gemüse, Getreide, Hülsenfrüchte/Bohnen, Samen, Nüsse, Pflanzenkäse, Pflanzenmilch, Pflanzenvleisch, pflanzliche Öle usw.	**Keine Produkte vom Tier** z. B. kein Fleisch oder Fisch; keine Eier, keine Milch und Milchprodukte; kein Käse und Butter; keinen Honig usw.

*

Bitte wählen Sie nur eine der folgenden Antworten aus:

◯ Mischkost (ich esse alles)

◯ Vegetarisch (pflanzlich, lakto-ovo-vegetarisch)

◯ Vegan (rein pflanzlich)

1.1 Gibt es mindestens 1 vegetarische Speise am Buffet (Jause) bzw. in der Kantine (Menü am Mittagstisch)? *

Beantworten Sie diese Frage nur, wenn folgende Bedingungen erfüllt sind:
Antwort war 'Vegetarisch (pflanzlich, lakto-ovo-vegetarisch)' bei Frage '38 [ErnaehrAkt]' (1. Wie ernähren Sie sich aktuell?Definition Ernährungsformen in Anlehnung an die weltweit größte Fachgesellschaft für Ernährung, Academy of Nutrition and Dietetics (AND, 2015/2016):)

Bitte wählen Sie nur eine der folgenden Antworten aus:

◯ Ja

◯ Nein

◯ Weiß nicht

1.2 Gibt es mindestens 1 vegane Speise am Buffet (Jause) bzw. in der Kantine (Menü am Mittagstisch)? *

Beantworten Sie diese Frage nur, wenn folgende Bedingungen erfüllt sind:
Antwort war 'Vegan (rein pflanzlich)' bei Frage '38 [ErnaehrAkt]' (1. Wie ernähren Sie sich aktuell?Definition Ernährungsformen in Anlehnung an die weltweit größte Fachgesellschaft für Ernährung, Academy of Nutrition and Dietetics (AND, 2015/2016):)

Bitte wählen Sie nur eine der folgenden Antworten aus:

- ◯ Ja
- ◯ Nein
- ◯ Weiß nicht

2. Wie lange ernähren Sie sich schon so? *

Bitte wählen Sie nur eine der folgenden Antworten aus:

- ◯ Schon immer, mein Leben lang
- ◯ ≤ 0,5 Jahr (1 – 6 Monate)
- ◯ 1 Jahr
- ◯ 2 Jahre
- ◯ 3 Jahre
- ◯ 4 Jahre
- ◯ 5 Jahre
- ◯ 6 – 9 Jahre
- ◯ ≥ 10 Jahre
- ◯ Sonstiges: ____________

3. Das ist mein Beweggrund für meine aktuelle Ernährung: *

Bitte wählen Sie die zutreffende Antwort für jeden Punkt aus:

	Ich stimme nicht zu 0	1	2	3	4	Ich stimme zu 5
**Gesundheit/Wohlbefinden (inkl. Gewichtsreduktion)**	○	○	○	○	○	○
**Sportliche Leistung**	○	○	○	○	○	○
**Tierschutz (Tierethik)**	○	○	○	○	○	○
**Klimaschutz**	○	○	○	○	○	○
**Umweltschutz (ökologische Aspekte)**	○	○	○	○	○	○
**Globale Ressourcenverteilung**	○	○	○	○	○	○
**Welthunger (soziale Aspekte, z. B. Land, Nahrung, etc.)**	○	○	○	○	○	○
**Religion/Spiritualität**	○	○	○	○	○	○
**Gewohnheit /Erziehung/Tradition**	○	○	○	○	○	○
**Geschmack/Genuss**	○	○	○	○	○	○
**Lebensmittelskandale, Lebensmittelsicherheit (z. B. Hygiene, Schadstoffe etc.)**	○	○	○	○	○	○
**Qualität der Nahrungsmittel (z. B. biologischer Anbau)**	○	○	○	○	○	○

	Ich stimme nicht zu<br>0	1	2	3	4	Ich stimme zu<br>5
**Epidemien, Pandemien (z. B. aktuelle COVID-19 Situation)**	○	○	○	○	○	○
**Preis von Nahrungsmitteln (z. B. billig, preiswert – finanzielle/ökonomische Aspekte)**	○	○	○	○	○	○
**Eltern/Geschwister /Familie**	○	○	○	○	○	○
**Freunde**	○	○	○	○	○	○
**(Studien-, Arbeits-)Kollegen/Bekannte**	○	○	○	○	○	○
**Vorbilder (z. B. Sportler, Popstars, Schauspieler)**	○	○	○	○	○	○
**Bestimmter Lifestyle (chic, „in“, dabei-sein, z. B. Medien, Promis, Veggie-Boom)**	○	○	○	○	○	○
**Andere Motive/Beweggründe**	○	○	○	○	○	○

TEIL C – ERNÄHRUNG

3a.1 Das ist mein Beweggrund für meine aktuelle Ernährung:

Bestimmter Lifestyle (chic, „in“, dabei-sein, z. B. Medien, Promis, Veggie-Boom) *

Beantworten Sie diese Frage nur, wenn folgende Bedingungen erfüllt sind:
Antwort war '

Ich stimme zu
5

' *oder* '

4

' *oder* '

3

' bei Frage '42 [BewegGERL1]' (3. Das ist mein Beweggrund für meine aktuelle Ernährung: (Bestimmter Lifestyle (chic, „in“, dabei-sein, z. B. Medien, Promis, Veggie-Boom)))

Bitte geben Sie Ihre Antwort hier ein:

3b.1 Das ist mein Beweggrund für meine aktuelle Ernährung:

Andere Motive/Beweggründe *

Beantworten Sie diese Frage nur, wenn folgende Bedingungen erfüllt sind:
Antwort war '

4

' *oder* '

Ich stimme zu
5

' *oder* '

3

' bei Frage '42 [BewegGERL1]' (3. Das ist mein Beweggrund für meine aktuelle Ernährung: (Andere Motive/Beweggründe))

Bitte geben Sie Ihre Antwort hier ein:

3.1 Meine Top-3 Motive für meine aktuelle Ernährung.

Wichtig!
Bitte nehmen Sie eine Top-3-Reihung für Ihre 3 Haupt-Motive für Ihre aktuelle Ernährung vor – Reihung mit absteigender Bedeutung/Gewichtung Top 1-2-3: 1 für das wichtigste Motiv, 2 für das 2./zweit-wichtigste Motiv, 3 für das 3./dritt-wichtigste Motiv. *

❗ Bitte wählen Sie 3 Antworten aus.
Bitte nummerieren Sie jede Box in der Reihenfolge Ihrer Präferenz, beginnen mit 1 bis 20
Bitte wählen Sie mindestens 3 Einträge aus.
Bitte wähle Sie nicht mehr als 3 Einträge aus.

[ ] Gesundheit/Wohlbefinden (inkl. Gewichtsreduktion)

[ ] Sportliche Leistung

[ ] Tierschutz (Tierethik)

[ ] Klimaschutz

[ ] Umweltschutz (ökologische Aspekte)

[ ] Globale Ressourcenverteilung

[ ] Welthunger (soziale Aspekte, z. B. Land, Nahrung, etc.)

[ ] Religion/Spiritualität

[ ] Gewohnheit /Erziehung/Tradition

[ ] Geschmack/Genuss

[ ] Lebensmittelskandale, Lebensmittelsicherheit (z. B. Hygiene, Schadstoffe etc.)

[ ] Qualität der Nahrungsmittel (z. B. biologischer Anbau)

Epidemien, Pandemien (z. B. aktuelle COVID-19 Situation)

Preis von Nahrungsmitteln (z. B. billig, preiswert – finanzielle/ökonomische Aspekte)

Eltern/Geschwister/Familie

Freunde

(Studien-, Arbeits-)Kollegen/Bekannte

Vorbilder (z. B. Sportler, Popstars, Schauspieler)

Bestimmter Lifestyle (chic, „in“, dabei-sein, z. B. Medien, Promis, Veggie-Boom)

Andere Motive/Beweggründe

4. Wieviel trinken Sie pro Tag (Aufstehen/Frühstück bis zum Schlafen-Gehen)?

Anmerkung:
0,25 Liter (bzw. 250 ml) entspricht z. B. 1 großen Tasse Tee; 0,5 Liter (bzw. 500 ml) entspricht z. B. 1 kleinen PET-Flasche Eistee oder 1 großem Glas Apfelsaft. *

Bitte wählen Sie nur eine der folgenden Antworten aus:

- weniger als 1 Liter/Tag
- zwischen 1 – 1,5 Liter/Tag
- zwischen 1,5 – 2 Liter/Tag
- zwischen 2 – 2,5 Liter/Tag
- mehr als 2,5 Liter/Tag

5. Was trinken Sie am häufigsten, z. B. täglich? *

Bitte wählen Sie nur eine der folgenden Antworten aus:

◯ Wasser (Leitungswasser, Mineralwasser, Sodawasser)

◯ Fruchtsäfte (verdünnt und unverdünnt, z. B. Apfelsaft, Orangensaft)

◯ Verdünnungssäfte (z. B. aus Himbeersirup, Holundersirup)

◯ Soft-Drinks (zuckerhaltige Getränke inkl. Light- und Zero-Produkte wie z. B. Eistee, Cola, Fanta, Sprite, Almdudler, usw.)

◯ Tee (heiß oder kalt)

◯ Kakao/heiße Schokolade

◯ Kaffee

◯ Energy Drinks (z. B. Red Bull, Flying Horse, etc.)

◯ Anderes: ____________________

6. Essen Sie täglich Obst? *

Bitte wählen Sie nur eine der folgenden Antworten aus:

◯ Ja

◯ Nein

6.1a Wie viele Portionen Obst täglich?

Anmerkung:
Die eigene Hand ist das Maß für 1 Portion Obst; d.h. 1 Portion Obst entspricht jener Menge, welche in die eigene Hand passt. *

Beantworten Sie diese Frage nur, wenn folgende Bedingungen erfüllt sind:
Antwort war 'Ja' bei Frage '49 [TaeglObst]' (6. Essen Sie täglich Obst?)

Bitte geben Sie Ihre Antwort hier ein:

[ ]

Portionen pro Tag

6.1b Wie oft essen Sie Obst? *

Beantworten Sie diese Frage nur, wenn folgende Bedingungen erfüllt sind:
Antwort war 'Nein' bei Frage '49 [TaeglObst]' (6. Essen Sie täglich Obst?)

Bitte wählen Sie nur eine der folgenden Antworten aus:

- ○ 1mal pro Woche
- ○ 2mal pro Woche
- ○ 3mal pro Woche
- ○ 4mal pro Woche
- ○ 5mal pro Woche
- ○ 6mal pro Woche
- ○ Seltener: wie oft? [ ]

7. Essen Sie täglich Gemüse? *

Bitte wählen Sie nur eine der folgenden Antworten aus:

- ◯ Ja
- ◯ Nein

7.1a Wie viele Portionen Gemüse täglich?

Anmerkung:
Die eigene Hand ist das Maß für 1 Portion Gemüse; d.h. 1 Portion Gemüse entspricht jener Menge, welche in die eigene Hand passt. *

Beantworten Sie diese Frage nur, wenn folgende Bedingungen erfüllt sind:
Antwort war 'Ja' bei Frage '52 [TeaglGemuese]' (7. Essen Sie täglich Gemüse?)

Bitte geben Sie Ihre Antwort hier ein:

[ ]

Portionen pro Tag

7.1b Wie oft essen Sie Gemüse? *

Beantworten Sie diese Frage nur, wenn folgende Bedingungen erfüllt sind:
Antwort war 'Nein' bei Frage '52 [TeaglGemuese]' (7. Essen Sie täglich Gemüse?)

Bitte wählen Sie nur eine der folgenden Antworten aus:

- ◯ 1mal pro Woche
- ◯ 2mal pro Woche
- ◯ 3mal pro Woche
- ◯ 4mal pro Woche
- ◯ 5mal pro Woche
- ◯ 6mal pro Woche
- ◯ Seltener: wie oft? ____________

8. Welche Mahlzeiten nehmen Sie in der Regel täglich zu sich? *

Bitte wählen Sie alle zutreffenden Antworten aus:

- ☐ Frühstück
- ☐ Mittagessen
- ☐ Abendessen
- ☐ Jause bzw. Zwischenmahlzeit oder Snacks

8.1 Wann nehmen Sie Ihre Jause bzw. Zwischenmahlzeit oder Snacks zu sich? *

Beantworten Sie diese Frage nur, wenn folgende Bedingungen erfüllt sind:
Antwort war bei Frage '55 [WelcheMahlzTaegl]' (8. Welche Mahlzeiten nehmen Sie in der Regel täglich zu sich?)

Bitte wählen Sie alle zutreffenden Antworten aus:

- [ ] Vormittags
- [ ] Nachmittags
- [ ] Nachts

9. Wie schätzen Sie die Verteilung der Hauptnährstoffe Kohlenhydrate, Protein (Eiweiß) und Fett (jeweils in %) gemessen an Ihrer Tagesenergiezufuhr (100 %) ein?

Anmerkung:
Die Gesamtsumme Ihrer Eingabe aus allen 3 Feldern darf nicht größer als 100 oder kleiner als 100 sein, d. h. muss insgesamt exakt 100 ergeben.

Beispiel:
45 % Kohlenhydrate + 35 % Protein + 20 % Fett = 100 % Ihrer Tagesenergie *

❗ Die Summe muss gleich 100 sein

❗ Nur ganzzahlige Werte können in diese Felder eingegeben werden.

Bitte geben Sie Ihre Antwort(en) hier ein:

Kohlenhydrate

Protein

Fett

TEIL D – GESUNDHEIT & WOHLBEFINDEN

Die Fragen beziehen sich NICHT ausschließlich bzw. sind NICHT limitiert auf den COVID-19 Lockdown (03-05/2020 bzw. 11/2020-02/2021).

1. Wie – denken Sie – ist Bewegung & Sport gesund und fördert Ihre Gesundheit?

Definition „Bewegung & Sport“:
Bewegungs- und/oder Sport-Aktivität ... z. B. Wandern oder Gartenarbeit uvm.

- *Mindestens 150 min/Woche moderate und/oder mindestens 75 min/Woche hohe Intensität, bei ...*
- *Erhöhtem Herzschlag/erhöhter Herzfrequenz, und*
- *Erhöhter Atmung/Atemfrequenz, und*
- *Erhöhter Körpertemperatur aufgrund von Bewegungs- und/oder Sport-Aktivität (vgl. nicht Sauna oder Solarium!), z. B. mir wir warm/heiß, ich schwitze, ich glühe etc.*

Beispiele:
Bewegungsaktivität, z. B. Gartenarbeit, Spazieren gehen ohne/mit Hund, etc.
Sportaktivität, z. B. Laufen/Jogging, Radfahren, Schwimmen, Schifahren, Langlaufen, etc.

1.1 Häufigkeit: An wievielen Tagen pro Woche – denken Sie – ist Bewegung & Sport gesund und fördert Ihre Gesundheit? *

Bitte wählen Sie nur eine der folgenden Antworten aus:

- ◯ Nie (kein mal pro Woche)
- ◯ Ab und zu, gelegentlich
- ◯ Regelmäßig
- ◯ Sonstiges: ________

1.1a Ab und zu, gelegentlich *

Beantworten Sie diese Frage nur, wenn folgende Bedingungen erfüllt sind:
Antwort war 'Ab und zu, gelegentlich' bei Frage '59 [SportProWocheTage]' (1.1 Häufigkeit: An wievielen Tagen pro Woche – denken Sie – ist Bewegung & Sport gesund und fördert Ihre Gesundheit?)

Bitte wählen Sie nur eine der folgenden Antworten aus:

- ◯ 3mal pro Monat
- ◯ Situationsspezifisch

1.1a.1 Situationsspezifisch *

Beantworten Sie diese Frage nur, wenn folgende Bedingungen erfüllt sind:
Antwort war 'Situationsspezifisch' bei Frage '60 [SportAbUndZu]' (1.1a Ab und zu, gelegentlich)

Bitte wählen Sie alle zutreffenden Antworten aus:

- ☐ An verlängerten Wochenenden
- ☐ Urlaub (z. B. in den Ferien)
- ☐ Saisonal (Winter vs. Sommer)
- ☐ Sonstiges

1.1a.1a Sonstiges *

Beantworten Sie diese Frage nur, wenn folgende Bedingungen erfüllt sind:
Antwort war bei Frage '61 [SportSituationssp]' (1.1a.1 Situationsspezifisch)

Bitte geben Sie Ihre Antwort hier ein:

1b.1 Regelmäßig *

Beantworten Sie diese Frage nur, wenn folgende Bedingungen erfüllt sind:
Antwort war 'Regelmäßig' bei Frage '59 [SportProWocheTage]' (1.1 Häufigkeit: An wievielen Tagen pro Woche – denken Sie – ist Bewegung & Sport gesund und fördert Ihre Gesundheit?)

Bitte wählen Sie nur eine der folgenden Antworten aus:

- ◯ Täglich (7mal pro Woche)
- ◯ 6mal pro Woche
- ◯ 5mal pro Woche
- ◯ 4mal pro Woche
- ◯ 3mal pro Woche
- ◯ 2mal pro Woche
- ◯ 1mal pro Woche

1.2 Dauer: Wie lange– denken Sie – soll Bewegung & Sport dauern, damit es gesund und förderlich für Ihre Gesundheit ist? *

Bitte wählen Sie nur eine der folgenden Antworten aus:

- ◯ pro Woche
- ◯ pro Tag

1.2.1 Dauer pro Woche *

Beantworten Sie diese Frage nur, wenn folgende Bedingungen erfüllt sind:
Antwort war 'pro Woche' bei Frage '64 [WieLangeSport]' (1.2 Dauer: Wie lange– denken Sie – soll Bewegung & Sport dauern, damit es gesund und förderlich für Ihre Gesundheit ist?)

❗ Bitte wählen Sie eine der folgenden Antworten:
Bitte wählen Sie nur eine der folgenden Antworten aus:

- ◯ 10 min pro Woche reichen aus
- ◯ 20 min pro Woche
- ◯ 30 min pro Woche
- ◯ 40 min pro Woche
- ◯ 50 min pro Woche
- ◯ 60 min pro Woche
- ◯ 75 min bzw. 1 ¼ Stunden pro Woche
- ◯ 90 – 120 min bzw. 1 ½ – 2 Stunden pro Woche
- ◯ 120 – 180 min bzw. 2 – 3 Stunden pro Woche
- ◯ 180 – 240 min bzw. 3 – 4 Stunden pro Woche
- ◯ 240 – 300 min bzw. 4 – 5 Stunden pro Woche
- ◯ > 300 min bzw. mehr als 5 Stunden pro Woche:

1.2.2 Dauer pro Tag *

Beantworten Sie diese Frage nur, wenn folgende Bedingungen erfüllt sind:
Antwort war 'pro Tag' bei Frage '64 [WieLangeSport]' (1.2 Dauer: Wie lange– denken Sie – soll Bewegung & Sport dauern, damit es gesund und förderlich für Ihre Gesundheit ist?)

Bitte wählen Sie nur eine der folgenden Antworten aus:

- ◯ 10 min pro Tag reichen aus
- ◯ 20 min pro Tag
- ◯ 30 min pro Tag
- ◯ 40 min pro Tag
- ◯ 50 min pro Tag
- ◯ 60 min pro Tag
- ◯ 75 min bzw. 1 ¼ Stunden pro Tag
- ◯ 90 min bzw. 1 ½ Stunden pro Tag
- ◯ 1 ¾ Stunden pro Tag
- ◯ 120 min bzw. 2 Stunden pro Tag
- ◯ > 120 min bzw. mehr als 2 Stunden pro Tag:

1.3 Intensität: Wie intensiv – denken Sie – soll *eine Einheit* Bewegung & Sport sein, damit es gesund und förderlich für Ihre Gesundheit ist?

Definition:

- *Niedere Intensität (locker, z. B. Jogging, Gartenarbeit) – Ich kann während der Bewegungs- & Sportaktivität noch gut sprechen & mich unterhalten!*
- *Moderate bzw. mittlere Intensität (gefordert, nicht zu locker/nicht zu anstrengend, z. B. Schwimmen) – Ich kann der Bewegungs- & Sportaktivität gerade noch sprechen & mich unterhalten!*
- *Hohe Intsentität (anstrengend, z. B. Mountainbiking) – Ich kann während der Bewegungs- & Sportaktivität nicht mehr sprechen & mich unterhalten!*

*

Bitte wählen Sie nur eine der folgenden Antworten aus:

◯ Niedere Intensität der Bewegungs- & Sportaktivität (locker)

◯ Moderate bzw. mittlere Intensität der Bewegungs- & Sportaktivität (gefordert, nicht zu locker/nicht zu anstrengend)

◯ Hohe Intensität der Bewegungs- & Sportaktivität (anstrengend)

2. Welche Nahrungsmittel – denken Sie – sind gesund und fördern Ihre Gesundheit? *

Bitte wählen Sie alle zutreffenden Antworten aus:

☐ Tierische Nahrungsmittel

☐ Pflanzliche Nahrungsmittel

☐ Nahrungsergänzungsmittel bzw. Supplemente

2.1 Tierische Nahrungsmittel *

Beantworten Sie diese Frage nur, wenn folgende Bedingungen erfüllt sind:
Antwort war bei Frage '68 [NahruFoerdGesundheit]' (2. Welche Nahrungsmittel – denken Sie – sind gesund und fördern Ihre Gesundheit?)

Bitte wählen Sie alle zutreffenden Antworten aus:

- [ ] Fleisch und Wurst, z. B. Schnitzel, Streichwurst, Fleischkäse/Leberkäse, Frankfurter Würstchen, Salami, Schinken, etc.
- [ ] Fisch und sog. „Meeresfrüchte“, z. B. Muscheln, Scampi, Fischöl, etc.
- [ ] Milch und Milchprodukte, z. B. Käse, Butter, Joghurt, Topfen/Quark, Sahne, etc.
- [ ] Eier
- [ ] Andere tierische Nahrungsmittel

Andere tierische Nahrungsmittel: *

Beantworten Sie diese Frage nur, wenn folgende Bedingungen erfüllt sind:
Antwort war bei Frage '69 [TierischeNahrungsm]' (2.1 Tierische Nahrungsmittel)

Bitte geben Sie Ihre Antwort hier ein:

2.2 Pflanzliche Nahrungsmittel *

Beantworten Sie diese Frage nur, wenn folgende Bedingungen erfüllt sind:
Antwort war bei Frage '68 [NahruFoerdGesundheit]' (2. Welche Nahrungsmittel – denken Sie – sind gesund und fördern Ihre Gesundheit?)

Bitte wählen Sie alle zutreffenden Antworten aus:

- [ ] Obst
- [ ] Gemüse
- [ ] Getreideprodukte, z. B. Brot, Nudeln, Reis, Müsli, etc.
- [ ] Kartoffeln und Hülsenfrüchte, z. B. Bohnen, Erbsen, etc.
- [ ] Andere pflanzliche Nahrungsmittel (z. B. pflanzliche Alternativen wie Vleisch, Visch, Sojamilch oder Ähnliches)

Andere pflanzliche Nahrungsmittel: *

Beantworten Sie diese Frage nur, wenn folgende Bedingungen erfüllt sind:
Antwort war bei Frage '71 [PflanzlicheNahrungsm]' (2.2 Pflanzliche Nahrungsmittel)

Bitte geben Sie Ihre Antwort hier ein:

2.3 Nahrungsergänzungsmittel bzw. Supplemente in Form von Tabletten, Pillen oder Pulver (in alphabetischer Reihenfolge)

- zur täglichen Zufuhr und adäquaten Versorgung von z. B. Vitaminen, Mineralstoffen und Spurenelementen, Protein, Ballaststoffe, Multivitamine, Bontalicals, Phytonutrients etc.
- zur gelegentlichen Kompensation von Unterversorgung und/oder Mängeln bzw. bei erhöhtem Bedarf wie z. B. bei Krankheit oder wenn man viel Sport treibt
- insbesondere sog. kritischer Nährstoffe bzw. Nährstoffen bzgl. häufig festgestellter Unterversorgung und/oder festgestellten Defiziten

*

Beantworten Sie diese Frage nur, wenn folgende Bedingungen erfüllt sind:
Antwort war 'Nahrungsergänzungsmittel bzw. Supplemente' bei Frage '68 [NahruFoerdGesundheit]' (2. Welche Nahrungsmittel – denken Sie – sind gesund und fördern Ihre Gesundheit?)

Bitte wählen Sie nur eine der folgenden Antworten aus:

- ◯ Ja
- ◯ Nein
- ◯ Weiß nicht

2.3.1 Zufuhr Nahrungsergänzungsmittel bzw. Supplemente *

Beantworten Sie diese Frage nur, wenn folgende Bedingungen erfüllt sind:
Antwort war 'Ja' bei Frage '73 [NahrungsergSuppl]' (2.3 Nahrungsergänzungsmittel bzw. Supplemente in Form von Tabletten, Pillen oder Pulver (in alphabetischer Reihenfolge) zur täglichen Zufuhr und adäquaten Versorgung von z. B. Vitaminen, Mineralstoffen und Spurenelementen, Protein, Ballaststoffe, Multivitamine, Bontalicals, Phytonutrients etc. zur gelegentlichen Kompensation von Unterversorgung und/oder Mängeln bzw. bei erhöhtem Bedarf wie z. B. bei Krankheit oder wenn man viel Sport treibt insbesondere sog. kritischer Nährstoffe bzw. Nährstoffen bzgl. häufig festgestellter Unterversorgung und/oder festgestellten Defiziten)

Bitte wählen Sie nur eine der folgenden Antworten aus:

- ◯ Täglich
- ◯ Manchmal

2.3.1a Zufuhr Nahrungsergänzungsmittel bzw. Supplemente *

Beantworten Sie diese Frage nur, wenn folgende Bedingungen erfüllt sind:
Antwort war 'Ja' bei Frage '73 [NahrungsergSuppl]' (2.3 Nahrungsergänzungsmittel bzw. Supplemente in Form von Tabletten, Pillen oder Pulver (in alphabetischer Reihenfolge) zur täglichen Zufuhr und adäquaten Versorgung von z. B. Vitaminen, Mineralstoffen und Spurenelementen, Protein, Ballaststoffe, Multivitamine, Bontalicals, Phytonutrients etc. zur gelegentlichen Kompensation von Unterversorgung und/oder Mängeln bzw. bei erhöhtem Bedarf wie z. B. bei Krankheit oder wenn man viel Sport treibt insbesondere sog. kritischer Nährstoffe bzw. Nährstoffen bzgl. häufig festgestellter Unterversorgung und/oder festgestellten Defiziten)

Bitte wählen Sie alle zutreffenden Antworten aus:

- ☐ Ballaststoffe
- ☐ Eisen
- ☐ Folsäure
- ☐ Jod
- ☐ Kalzium
- ☐ Kohlenhydrate
- ☐ Kupfer
- ☐ Omega-3-Fettsäuren
- ☐ Protein
- ☐ Vitamin B12
- ☐ Vitamin C
- ☐ Vitamin D
- ☐ Vitamin E
- ☐ Zink
- ☐ Andere Nahrungsergänzungsmittel/Supplemente

Andere Nahrungsergänzungsmittel/Supplemente: *

Beantworten Sie diese Frage nur, wenn folgende Bedingungen erfüllt sind:
Antwort war bei Frage '75 [Supplemente]' (2.3.1a Zufuhr Nahrungsergänzungsmittel bzw. Supplemente)

Bitte geben Sie Ihre Antwort hier ein:

TEIL D – GESUNDHEIT & WOHLBEFINDEN

2.4 Welche Nahrungsmittel – denken Sie – sind gesund und fördern Ihre Gesundheit?

Wichtig!
Bitte nehmen Sie auch eine Top-3-Reihung vor – Reihung mit absteigender Bedeutung/Gewichtung Top 1-2-3: 1 für den wichtigsten Faktor, 2 für den 2./zweit-wichtigsten Faktor, 3 für den 3./dritt-wichtigsten Faktor. *

❗ Bitte wählen Sie zwischen 1 und 3 Antworten aus.
Bitte nummerieren Sie jede Box in der Reihenfolge Ihrer Präferenz, beginnen mit 1 bis 25
Bitte wählen Sie mindestens 1 Einträge aus.

☐ Fleisch und Wurst, z. B. Schnitzel, Streichwurst, Fleischkäse/Leberkäse, Frankfurter Würstchen, Salami, Schinken, etc.

☐ Fisch und sog. „Meeresfrüchte", z. B. Muscheln, Scampi, Fischöl, etc.

☐ Milch und Milchprodukte, z. B. Käse, Butter, Joghurt, Topfen/Quark, Sahne, etc.

☐ Eier

☐ Andere tierische Nahrungsmittel

☐ Obst

☐ Gemüse

☐ Getreideprodukte, z. B. Brot, Nudeln, Reis, Müsli, etc.

☐ Kartoffeln und Hülsenfrüchte, z. B. Bohnen, Erbsen, etc.

☐ Andere pflanzliche Nahrungsmittel (z. B. pflanzliche Alternativen wie Vleisch, Visch, Sojamilch oder Ähnliches)

☐ Ballaststoffe

- [ ] Eisen
- [ ] Folsäure
- [ ] Jod
- [ ] Kalzium
- [ ] Kohlenhydrate
- [ ] Kupfer
- [ ] Omega-3-Fettsäuren
- [ ] Protein
- [ ] Vitamin B12
- [ ] Vitamin C
- [ ] Vitamin D
- [ ] Vitamin E
- [ ] Zink
- [ ] Andere Nahrungsergänzungsmittel/Supplemente

3. Ihre Schlafdauer und Schlafqualität?

3.1 Wie vielen Stunden schalfen Sie pro Nacht (im Durschschnitt)? *

Bitte wählen Sie die zutreffende Antwort für jeden Punkt aus:

	Stunden	Minuten
	[illegible]	[illegible]
**an Arbeitstagen**		
**an Wochenenden (Samstag, Sonntag) bzw. Feiertagen**		

3.2.1 Fühlen Sie sich an Arbeitstagen am Morgen grundsätzlich ausgeschlafen und erholt? *

Bitte wählen Sie nur eine der folgenden Antworten aus:

- Ja
- Eher ja
- Eher nein
- Nein
- Anderes (z. B. Schlafstörungen etc.):

3.2.2 Fühlen Sie sich an Wochenenden am Morgen grundsätzlich ausgeschlafen und erholt? *

Bitte wählen Sie nur eine der folgenden Antworten aus:

- ◯ Ja
- ◯ Eher ja
- ◯ Eher nein
- ◯ Nein
- ◯ Anderes (z. B. Schlafstörungen etc.):

4. Welche anderen Faktoren (neben Bewegung und Ernährung) – denken Sie – sind gesund und fördern Ihre Gesundheit? *

Bitte wählen Sie alle zutreffenden Antworten aus:

- ☐ Erholsamer Schlaf
- ☐ Bewältigung von Druck und Stress beruflich und privat
- ☐ Vermeidung von Genussmitteln (z. B. Energydrinks, Alkohol etc.)
- ☐ Positive Beziehungen beruflich und privat
- ☐ Nahrungsergänzungsmittel/Supplemente
- ☐ Andere (einzelne Faktoren, z. B. sinnerfüllte berufliche Tätigkeit)::

4.1 Welche Faktoren – denken Sie – sind die Top 3 Faktoren, welche gesund sind und Ihre Gesundheit fördern?

Wichtig!
Bitte nehmen Sie eine Top-3-Reihung vor – Reihung mit absteigender Bedeutung/Gewichtung Top 1-2-3: 1 für den wichtigsten Faktor, 2 für den 2./zweit-wichtigsten Faktor, 3 für den 3./dritt-wichtigsten Faktor. *

❗ Alle Ihre Antworten müssen unterschiedlich sein, und müssen zugeordnet sein.

❗ Bitte wählen Sie 3 Antworten aus.

Bitte nummerieren Sie jede Box in der Reihenfolge Ihrer Präferenz, beginnen mit 1 bis 9

Bitte wählen Sie mindestens 3 Einträge aus.

Bitte wähle Sie nicht mehr als 3 Einträge aus.

☐ Bewegung & Sport

☐ Ernährung

☐ Schlaf

☐ Druck & Stress

☐ Genussmittel

☐ Beziehungen

☐ Nahrungsergänzungsmittel/Supplemente

☐ Andere (einzelne Faktoren, z. B. sinnerfüllte berufliche Tätigkeit)

☐ Sonstiges (Faktoren in Kombination)

4.1a Welche sonstigen Faktoren in Kombination? *

Beantworten Sie diese Frage nur, wenn folgende Bedingungen erfüllt sind:
-------- Scenario 1 --------
Antwort war 'Sonstiges (Faktoren in Kombination)' bei Frage '86 [GesFaktorReihung]' (4.1 Welche Faktoren – denken Sie – sind die Top 3 Faktoren, welche gesund sind und Ihre Gesundheit fördern?Wichtig!Bitte nehmen Sie eine Top-3-Reihung vor – Reihung mit absteigender Bedeutung/Gewichtung Top 1-2-3: 1 für den wichtigsten Faktor, 2 für den 2./zweit-wichtigsten Faktor, 3 für den 3./dritt-wichtigsten Faktor. (RANK 1))
-------- oder Scenario 2 --------
Antwort war 'Sonstiges (Faktoren in Kombination)' bei Frage '86 [GesFaktorReihung]' (4.1 Welche Faktoren – denken Sie – sind die Top 3 Faktoren, welche gesund sind und Ihre Gesundheit fördern?Wichtig!Bitte nehmen Sie eine Top-3-Reihung vor – Reihung mit absteigender Bedeutung/Gewichtung Top 1-2-3: 1 für den wichtigsten Faktor, 2 für den 2./zweit-wichtigsten Faktor, 3 für den 3./dritt-wichtigsten Faktor. (RANK 2))
-------- oder Scenario 3 --------
Antwort war 'Sonstiges (Faktoren in Kombination)' bei Frage '86 [GesFaktorReihung]' (4.1 Welche Faktoren – denken Sie – sind die Top 3 Faktoren, welche gesund sind und Ihre Gesundheit fördern?Wichtig!Bitte nehmen Sie eine Top-3-Reihung vor – Reihung mit absteigender Bedeutung/Gewichtung Top 1-2-3: 1 für den wichtigsten Faktor, 2 für den 2./zweit-wichtigsten Faktor, 3 für den 3./dritt-wichtigsten Faktor. (RANK 3))

Bitte geben Sie Ihre Antwort hier ein:

5. Leiden Sie an Lebensmittel-/Nahrungsmittel-Allergien oder -Intoleranzen? *

Bitte wählen Sie nur eine der folgenden Antworten aus:

- ○ Ja
- ○ Nein

5.1 Welche Lebensmittel-/Nahrungsmittel-Allergien oder -Intoleanzen haben Sie? *

Beantworten Sie diese Frage nur, wenn folgende Bedingungen erfüllt sind:
Antwort war 'Ja' bei Frage '88 [LebensmAllergien]' (5. Leiden Sie an Lebensmittel-/Nahrungsmittel-Allergien oder -Intoleranzen?)

Bitte geben Sie Ihre Antwort hier ein:

6. Leiden Sie an einer chronischen und/oder schweren Erkrankung? *

Bitte wählen Sie nur eine der folgenden Antworten aus:

- ○ Ja
- ○ Nein
- ○ Keine Angabe

7. Ihre Lebensqualität (lt. WHO-QOL-BREF26)?

Wichtig!
Bitte beurteilen Sie Ihre Lebensqualität, Ihre Gesundheit und andere Bereiche Ihres Lebens auf Basis der vergangenen 2 Wochen; überlegen Sie, wie Sie sich während der vergangenen 2 Wochen gefühlt haben, und kreuzen Sie das entsprechende Feld in der Skala an, das am ehesten für Sie zutrifft.

7.1.1 Wie haben Sie sich während der vergangenen 2 Wochen gefühlt? *

Bitte wählen Sie die zutreffende Antwort für jeden Punkt aus:

	Sehr schlecht	Schlecht	Mittelmäßig	Gut	Sehr gut
**Wie würden Sie Ihre Lebensqualität beurteilen?**	○	○	○	○	○

7.1.2 Wie haben Sie sich während der vergangenen 2 Wochen gefühlt? *

Bitte wählen Sie die zutreffende Antwort für jeden Punkt aus:

	Sehr unzufrieden	Unzufrieden	Weder noch	Zufrieden	Sehr zufrieden
**Wie zufrieden sind Sie mit Ihrer Gesundheit?**	○	○	○	○	○

7.2 Wie stark haben Sie während der vergangenen 2 Wochen bestimmte Dinge erlebt? *

Bitte wählen Sie die zutreffende Antwort für jeden Punkt aus:

	Überhaupt nicht	Ein wenig	Mittelmäßig	Ziemlich	Äußerst
**Wie stark werden Sie durch Schmerzen daran gehindert, notwendige Dinge zu tun?**	○	○	○	○	○
**Wie sehr sind Sie auf medizinische Behandlung angewiesen, um das tägliche Leben zu meistern?**	○	○	○	○	○
**Wie gut können Sie Ihr Leben genießen?**	○	○	○	○	○
**Betrachten Sie Ihr Leben als sinnvoll?**	○	○	○	○	○
**Wie gut können Sie sich konzentrieren?**	○	○	○	○	○
**Wie sicher fühlen Sie sich in Ihrem täglichen Leben?**	○	○	○	○	○
**Wie gesund sind die Umweltbedingungen in Ihrem Wohngebiet?**	○	○	○	○	○

7.3.1 In welchem Umfang haben Sie während der vergangenen 2 Wochen bestimmte Dinge erlebt oder waren in der Lage, bestimmte Dinge zu tun? *

Bitte wählen Sie die zutreffende Antwort für jeden Punkt aus:

	Überhaupt nicht	Eher nicht	Halbwegs	Überwiegend	Völlig
**Haben Sie genug Energie für das tägliche Leben?**	○	○	○	○	○
**Können Sie Ihr Aussehen akzeptieren?**	○	○	○	○	○
**Haben Sie genug Geld, um Ihre Bedürfnisse zu befriedigen?**	○	○	○	○	○
**Haben Sie Zugang zu den Informationen, die Sie für das tägliche Leben brauchen?**	○	○	○	○	○
**Haben Sie ausreichend Möglichkeiten zu Freizeitaktivitäten?**	○	○	○	○	○

7.3.2 In welchem Umfang haben Sie während der vergangenen 2 Wochen bestimmte Dinge erlebt oder waren in der Lage, bestimmte Dinge zu tun? *

Bitte wählen Sie die zutreffende Antwort für jeden Punkt aus:

	Sehr schlecht	Schlecht	Mittelmäßig	Gut	Sehr gut
**Wie gut können Sie sich fortbewegen?**	◯	◯	◯	◯	◯

7.4 Wie zufrieden, glücklich oder gut haben Sie sich während der vergangenen 2 Wochen hinsichtlich verschiedener Aspekte Ihres Lebens gefühlt? *

Bitte wählen Sie die zutreffende Antwort für jeden Punkt aus:

	Sehr unzufrieden	Unzufrieden	Weder noch	Zufrieden	Sehr zufrieden
**Wie zufrieden sind Sie mit Ihrem Schlaf?**	○	○	○	○	○
**Wie zufrieden sind Sie mit Ihrer Fähigkeit, alltägliche Dinge erledigen zu können?**	○	○	○	○	○
**Wie zufrieden sind Sie mit Ihrer Arbeitsfähigkeit?**	○	○	○	○	○
**Wie zufrieden sind Sie mit sich selbst?**	○	○	○	○	○
**Wie zufrieden sind Sie mit Ihren persönlichen Beziehungen?**	○	○	○	○	○
**Wie zufrieden sind Sie mit Ihrem Sexualleben?**	○	○	○	○	○
**Wie zufrieden sind Sie mit der Unterstützung durch Ihre Freunde?**	○	○	○	○	○
**Wie zufrieden sind Sie mit Ihren Wohnbedingungen?**	○	○	○	○	○
**Wie zufrieden sind Sie mit Ihren Möglichkeiten, Gesundheitsdienste in Anspruch nehmen zu können?**	○	○	○	○	○

	Sehr unzufrieden	Unzufrieden	Weder noch	Zufrieden	Sehr zufrieden
**Wie zufrieden sind Sie mit den Beförderungsmitteln, die Ihnen zur Verfügung stehen?**	◯	◯	◯	◯	◯

7.5 Wie oft haben sich während der vergangenen zwei Wochen bei Ihnen negative Gefühle eingestellt, wie zum Beispiel Angst oder Traurigkeit? *

Bitte wählen Sie die zutreffende Antwort für jeden Punkt aus:

	Niemals	Nicht oft	Zeitweilig	Oftmals	Immer
**Wie häufig haben Sie negative Gefühle wie Traurigkeit, Verzweiflung, Angst oder Depression?**	◯	◯	◯	◯	◯

TEIL E – COVID-19

Die Fragen beziehen sich ***ausschließlich*** auf den COVID-19 Lockdown (03-05/2020 bzw. 11/2020-02/2021).

1. Hat sich Ihr Bewegungs- und Sportverhalten *während* des COVID-19 Lockdowns verändert? *

Bitte wählen Sie nur eine der folgenden Antworten aus:

- ◯ Nein
- ◯ Ja, ich habe weniger Bewegung und Sport gemacht
- ◯ Ja, ich habe mehr Bewegung und Sport gemacht

2. Hat sich Ihr Ernährungsverhalten *während* des COVID-19 Lockdowns verändert? *

Bitte wählen Sie nur eine der folgenden Antworten aus:

- ◯ Nein
- ◯ Ja, ich habe mich gesünder ernährt
- ◯ Ja, ich habe mich weniger gesund ernährt

3. Hat sich Ihr durchschnittliches Körpergewicht *während* des COVID-19 Lockdowns geändert? *

Bitte wählen Sie nur eine der folgenden Antworten aus:

- ◯ Nein
- ◯ Ja, ich habe zugenommen
- ◯ Ja, ich habe abgenommen

3.1 Ja, ich habe zugenommen um etwa ... *

Beantworten Sie diese Frage nur, wenn folgende Bedingungen erfüllt sind:
Antwort war 'Ja, ich habe zugenommen ' bei Frage '101 [GewichtAenderCOV19]' (3. Hat sich Ihr durchschnittliches Körpergewicht während des COVID-19 Lockdowns geändert?)

❗ Ihre Antwort darf maximal 100 sein.
Bitte geben Sie Ihre Antwort hier ein:

[ ]

kg

3.2 Ja, ich habe abgenommen um etwa ... *

Beantworten Sie diese Frage nur, wenn folgende Bedingungen erfüllt sind:
Antwort war 'Ja, ich habe abgenommen ' bei Frage '101 [GewichtAenderCOV19]' (3. Hat sich Ihr durchschnittliches Körpergewicht während des COVID-19 Lockdowns geändert?)

❗ Ihre Antwort darf maximal 100 sein.
Bitte geben Sie Ihre Antwort hier ein:

[ ]

kg

4. Glauben Sie, dass regelmäßige Bewegung und Sport das Immunsystem stärken und sich so positiv auf eine COVID-19 Erkrankung auswirken können? *

Bitte wählen Sie nur eine der folgenden Antworten aus:

- ◯ Ja
- ◯ Nein
- ◯ Weiß nicht

5. Glauben Sie, dass eine bedarfsgerechte Ernährung das Immunsystem stärkt und sich so positiv auf eine COVID-19 Erkrankung auswirken kann? *

Bitte wählen Sie nur eine der folgenden Antworten aus:

- ◯ Ja
- ◯ Nein
- ◯ Weiß nicht

6. Hat Sie der COVID-19 Lockdown zu einer *nachhaltigen* Verhaltensänderung veranlasst?

6.1 Bewegung und Sport
Hat Sie der COVID-19 Lockdown zu einer *nachhaltigen* Verhaltensänderung veranlasst? *

Bitte wählen Sie nur eine der folgenden Antworten aus:

- ◯ Ja
- ◯ Nein

6.1.1 Bewegung und Sport *

Beantworten Sie diese Frage nur, wenn folgende Bedingungen erfüllt sind:
Antwort war 'Ja' bei Frage '107 [BewegSportCOV19]' (6.1 Bewegung und SportHat Sie der COVID-19 Lockdown zu einer nachhaltigen Verhaltensänderung veranlasst?)

Bitte wählen Sie alle zutreffenden Antworten aus:

- ☐ Ja, ich bin jetzt aktiver und betreibe mehr Bewegung und Sport
- ☐ Ja, ich habe neue Bewegungs- & Sportaktivitäten entdeckt

6.1.1a Bewegung und Sport
Ja, ich habe diese neuen Bewegungs- & Sportaktivitäten entdeckt:

Beantworten Sie diese Frage nur, wenn folgende Bedingungen erfüllt sind:
Antwort war bei Frage '108 [BewegSportJaCOV19]' (6.1.1 Bewegung und Sport)

Bitte geben Sie Ihre Antwort hier ein:

[   ]

6.2 Ernährung
Hat Sie der COVID-19 Lockdown zu einer *nachhaltigen* Verhaltensänderung veranlasst? *

Bitte wählen Sie nur eine der folgenden Antworten aus:

- ◯ Ja
- ◯ Nein

6.2.1 Ernährung *

Beantworten Sie diese Frage nur, wenn folgende Bedingungen erfüllt sind:
Antwort war 'Ja' bei Frage '110 [ERLCOV19]' (6.2 ErnährungHat Sie der COVID-19 Lockdown zu einer nachhaltigen Verhaltensänderung veranlasst?)

Bitte wählen Sie alle zutreffenden Antworten aus:

- ☐ Ja, ich ernähre mich jetzt gesünder
- ☐ Ja, ich habe neue bzw. andere Rezepte, Speisen, Nahrungs- & Lebensmittel entdeckt, nachhaltig eine andere Ernährungsform angenommen

6.2.1a Ernährung

Ich habe folgendes neu entdeckt – das ist jetzt anders als zuvor:

Beantworten Sie diese Frage nur, wenn folgende Bedingungen erfüllt sind:
Antwort war bei Frage '111 [ERLJaCOV19]' (6.2.1 Ernährung)

Bitte geben Sie Ihre Antwort hier ein:

[ ]

6.3 Bewusstes Einkaufs- & Konsumverhalten bzgl. klimafreundlicher Produkte
Hat Sie der COVID-19 Lockdown zu einer *nachhaltigen* Verhaltensänderung veranlasst? *

Bitte wählen Sie nur eine der folgenden Antworten aus:

- ◯ Ja
- ◯ Nein

6.3.1 Ja, ich kaufe und konsumiere jetzt bewusster bzgl. klimafreundlicher Produkte

Was ist jetzt anders als zuvor? *

Beantworten Sie diese Frage nur, wenn folgende Bedingungen erfüllt sind:
Antwort war 'Ja' bei Frage '113 [KonsumCOV19]' (6.3 Bewusstes Einkaufs- & Konsumverhalten bzgl. klimafreundlicher ProdukteHat Sie der COVID-19 Lockdown zu einer nachhaltigen Verhaltensänderung veranlasst?)

Bitte geben Sie Ihre Antwort hier ein:

6.4 Höhere Hilfsbereitschaft den Mitmenschen gegenüber
Hat Sie der COVID-19 Lockdown zu einer *nachhaltigen* Verhaltensänderung veranlasst? *

Bitte wählen Sie nur eine der folgenden Antworten aus:

- ◯ Ja
- ◯ Nein

6.4.1 Ja, ich bin jetzt insgesamt hilfsbereiter meinen Mitmenschen gegenüber

Was ist jetzt anders als zuvor? *

Beantworten Sie diese Frage nur, wenn folgende Bedingungen erfüllt sind:
Antwort war 'Ja' bei Frage '115 [HilfsbCOV19]' (6.4 Höhere Hilfsbereitschaft den Mitmenschen gegenüberHat Sie der COVID-19 Lockdown zu einer nachhaltigen Verhaltensänderung veranlasst?)

Bitte geben Sie Ihre Antwort hier ein:

6.5 Sonstige Verhaltensänderung(en) aufgrund des COVID-19 Lockdown?

Bitte geben Sie Ihre Antwort hier ein:

TEIL F – SONSTIGES zum Abschluss

Die Fragen beziehen sich NICHT ausschließlich bzw. sind NICHT limitiert auf den COVID-19 Lockdown (03-05/2020 bzw. 11/2020-02/2021).

1. Essen Sie folgende Lebensmittel?

Wichtig!
Mehrfachnennung möglich. *

Bitte wählen Sie alle zutreffenden Antworten aus:

- [ ] Obst
- [ ] Gemüse
- [ ] Getreideprodukte, z. B. Brot, Nudeln, Reis, Müsli, Cornflakes, Seitan, pflanzliche Alternativen (z. B. Vleisch, Visch, Sojamilch etc.) uvm.
- [ ] Kartoffeln und Hülsenfrüchte, z. B. Bohnen, Erbsen, Linsen, Tofu, Pommes Frites, etc.
- [ ] Fleisch und Wurst, z. B. Schnitzel, Streichwurst, Fleischkäse/Leberkäse, Frankfurter Würstchen, Salami, Schinken, etc.
- [ ] Fisch und sog. „Meeresfrüchte“, z. B. Muscheln, Scampi, Fischöl, etc.
- [ ] Milch und Milchprodukte, z. B. Käse, Butter, Joghurt, Topfen/Quark, Sahne, etc.
- [ ] Eier

2. Ihre Top 1-2-3 Freizeitaktivitäten?

Wichtig!
Mehrfachnennung möglich. Bitte nehmen Sie eine Top-3-Reihung für Ihre 3 wichtigsten Freizeitaktivitäten vor – Reihung mit absteigender Bedeutung/Gewichtung Top 1-2-3: 1 für die wichtigste Freizeitaktivität, 2 für die 2./zweit-wichtigste Freizeitaktivität, 3 für die 3./dritt-wichtigste Freizeitaktivität. *

❗ Alle Ihre Antworten müssen unterschiedlich sein, und müssen zugeordnet sein.

❗ Bitte wählen Sie 3 Antworten aus.

Bitte nummerieren Sie jede Box in der Reihenfolge Ihrer Präferenz, beginnen mit 1 bis 8

Bitte wählen Sie mindestens 3 Einträge aus.

Bitte wähle Sie nicht mehr als 3 Einträge aus.

☐ Fernsehen und Video-on-Demand (z. B. Netflix, Amazon usw.)

☐ Musik (z. B. hören, singen, Instrument spielen ...)

☐ Computer spielen/Internet surfen

☐ Sport treiben

☐ Lesen, Schreiben

☐ Freunde treffen (z. B. Bummel, Chillen, Shoppen gehen, etc.)

☐ Basteln, Malen, Handarbeiten

☐ Andere

2.1 Welche anderen Freizeitaktivitäten? *

Beantworten Sie diese Frage nur, wenn folgende Bedingungen erfüllt sind:
-------- Scenario 1 --------
Antwort war 'Andere' bei Frage '119 [TopFreizeitaktiv]' (2. Ihre Top 1-2-3 Freizeitaktivitäten?Wichtig!Mehrfachnennung möglich. Bitte nehmen Sie eine Top-3-Reihung für Ihre 3 wichtigsten Freizeitaktivitäten vor – Reihung mit absteigender Bedeutung/Gewichtung Top 1-2-3: 1 für die wichtigste Freizeitaktivität, 2 für die 2./zweit-wichtigste Freizeitaktivität, 3 für die 3./dritt-wichtigste Freizeitaktivität. (RANK 1))
-------- oder Scenario 2 --------
Antwort war 'Andere' bei Frage '119 [TopFreizeitaktiv]' (2. Ihre Top 1-2-3 Freizeitaktivitäten?Wichtig!Mehrfachnennung möglich. Bitte nehmen Sie eine Top-3-Reihung für Ihre 3 wichtigsten Freizeitaktivitäten vor – Reihung mit absteigender Bedeutung/Gewichtung Top 1-2-3: 1 für die wichtigste Freizeitaktivität, 2 für die 2./zweit-wichtigste Freizeitaktivität, 3 für die 3./dritt-wichtigste Freizeitaktivität. (RANK 2))
-------- oder Scenario 3 --------
Antwort war 'Andere' bei Frage '119 [TopFreizeitaktiv]' (2. Ihre Top 1-2-3 Freizeitaktivitäten?Wichtig!Mehrfachnennung möglich. Bitte nehmen Sie eine Top-3-Reihung für Ihre 3 wichtigsten Freizeitaktivitäten vor – Reihung mit absteigender Bedeutung/Gewichtung Top 1-2-3: 1 für die wichtigste Freizeitaktivität, 2 für die 2./zweit-wichtigste Freizeitaktivität, 3 für die 3./dritt-wichtigste Freizeitaktivität. (RANK 3))

Bitte geben Sie Ihre Antwort hier ein:

3. Rauchen Sie z. B. Zigaretten (auch e-Zigaretten, Pfeife, Zigarillo, Zigarre etc.)? *

Bitte wählen Sie nur eine der folgenden Antworten aus:

- ◯ Ja
- ◯ Nein

3.1 Wie oft rauchen Sie? *

Beantworten Sie diese Frage nur, wenn folgende Bedingungen erfüllt sind:
Antwort war 'Ja' bei Frage '121 [Rauchen]' (3. Rauchen Sie z. B. Zigaretten (auch e-Zigaretten, Pfeife, Zigarillo, Zigarre etc.)?)

Bitte wählen Sie nur eine der folgenden Antworten aus:

- ◯ Täglich
- ◯ Regelmäßig, aber nicht täglich
- ◯ gelegentlich

3.1a Wie viele Zigaretten rauchen Sie täglich? *

Beantworten Sie diese Frage nur, wenn folgende Bedingungen erfüllt sind:
Antwort war 'Täglich' bei Frage '122 [WieOftRauchen]' (3.1 Wie oft rauchen Sie?)

Bitte wählen Sie nur eine der folgenden Antworten aus:

- ◯ 1
- ◯ 2
- ◯ 3
- ◯ 4
- ◯ 5
- ◯ 6
- ◯ 7
- ◯ 8
- ◯ 9
- ◯ 10
- ◯ 11
- ◯ 12
- ◯ 13
- ◯ 14
- ◯ 15
- ◯ 16
- ◯ 17
- ◯ 18
- ◯ 19
- ◯ 20
- ◯ 21
- ◯ 22
- ◯ 23
- ◯ 24
- ◯ 25
- ◯ 26
- ◯ 27
- ◯ 28

- ○ 29
- ○ 30
- ○ 31
- ○ 32
- ○ 33
- ○ 34
- ○ 35
- ○ 36
- ○ 37
- ○ 38
- ○ 39
- ○ 40
- ○ 41
- ○ 42
- ○ 43
- ○ 44
- ○ 45
- ○ 46
- ○ 47
- ○ 48
- ○ 49
- ○ 50
- ○ 51
- ○ 52
- ○ 53
- ○ 54
- ○ 55
- ○ 56
- ○ 57
- ○ 58
- ○ 59
- ○ 60
- ○ 61

- ◯ 62
- ◯ 63
- ◯ 64
- ◯ 65
- ◯ 66
- ◯ 67
- ◯ 68
- ◯ 69
- ◯ 70
- ◯ 71
- ◯ 72
- ◯ 73
- ◯ 74
- ◯ 75
- ◯ 76
- ◯ 77
- ◯ 78
- ◯ 79
- ◯ 80
- ◯ 81
- ◯ 82
- ◯ 83
- ◯ 84
- ◯ 85
- ◯ 86
- ◯ 87
- ◯ 88
- ◯ 89
- ◯ 90
- ◯ 91
- ◯ 92
- ◯ 93
- ◯ 94

- ◯ 95
- ◯ 96
- ◯ 97
- ◯ 98
- ◯ 99
- ◯ 100

4. Trinken Sie Alkohol? *

Bitte wählen Sie nur eine der folgenden Antworten aus:

- ◯ Ja
- ◯ Nein

4.1 Was trinken Sie am häufigsten? *

Beantworten Sie diese Frage nur, wenn folgende Bedingungen erfüllt sind:
Antwort war 'Ja' bei Frage '124 [AlkoholTrinken]' (4. Trinken Sie Alkohol?)

Bitte wählen Sie nur eine der folgenden Antworten aus:

- ◯ Alkoholische Softgetränke (z. B. Alkopops, Radler, Sommerspritzer etc.)
- ◯ Bier
- ◯ Wein
- ◯ Schnaps bzw. harte Getränke
- ◯ Andere: ____________________

4.2 Wie oft trinken Sie Alkohol? *

Beantworten Sie diese Frage nur, wenn folgende Bedingungen erfüllt sind:
Antwort war 'Ja' bei Frage '124 [AlkoholTrinken]' (4. Trinken Sie Alkohol?)

Bitte wählen Sie nur eine der folgenden Antworten aus:

- ◯ Täglich
- ◯ Regelmäßig, aber nicht täglich
- ◯ gelegentlich

4.2.a Wie viel trinken Sie täglich? *

Beantworten Sie diese Frage nur, wenn folgende Bedingungen erfüllt sind:
Antwort war 'Täglich' bei Frage '126 [WieOftAlkohol]' (4.2 Wie oft trinken Sie Alkohol?)

❗ Kommentieren wenn eine Antwort gewählt wird
Bitte wählen Sie die zutreffenden Punkte aus und schreiben Sie einen Kommentar dazu:

- ☐ Alkoholische Softgetränke (z. B. Alkopops, Radler, Sommerspritzer etc.) (Beispiel: 0,2 oder 0,3 oder 0,5 oder 0,75, oder 1 Liter usw.)
- ☐ Bier (Beispiel: 0,2 oder 0,3 oder 0,5 oder 0,75, oder 1 Liter usw.)
- ☐ Wein (Beispiel: 1/8 oder ¼ oder ½ oder 0,75 oder 1 Liter usw.)
- ☐ Schnaps bzw. harte Getränke (Beispiel: 1 cl oder 2 cl oder 3 cl oder 4 cl oder 5 cl usw.)
- ☐ Andere

Übermittlung Ihres ausgefüllten Fragebogens:

Vielen Dank für die Beantwortung des Fragebogens.
